# Fast temporal dynamics and causal relevance of face processing in the human temporal cortex

**DOI:** 10.1038/s41467-020-14432-8

**Published:** 2020-01-31

**Authors:** Jessica Schrouff, Omri Raccah, Sori Baek, Vinitha Rangarajan, Sina Salehi, Janaina Mourão-Miranda, Zeinab Helili, Amy L. Daitch, Josef Parvizi

**Affiliations:** 1 Laboratory of Behavioral and Cognitive Neuroscience, Stanford University, Palo Alto, CA USA; 2 Computer Science Department, University College London, Gower street, London, WC1E6BT UK; 3 Department of Psychology, University of California, Berkeley, CA USA; 4 Department of Neurology and Neurological Sciences, Stanford University Medical Center, Palo Alto, CA USA

**Keywords:** Consciousness, Perception, Sensory processing, Social neuroscience

## Abstract

We measured the fast temporal dynamics of face processing simultaneously across the human temporal cortex (TC) using intracranial recordings in eight participants. We found sites with selective responses to faces clustered in the ventral TC, which responded increasingly strongly to marine animal, bird, mammal, and human faces. Both face-selective and face-active but non-selective sites showed a posterior to anterior gradient in response time and selectivity. A sparse model focusing on information from the human face-selective sites performed as well as, or better than, anatomically distributed models when discriminating faces from non-faces stimuli. Additionally, we identified the posterior fusiform site (pFUS) as causally the most relevant node for inducing distortion of conscious face processing by direct electrical stimulation. These findings support anatomically discrete but temporally distributed response profiles in the human brain and provide a new common ground for unifying the seemingly contradictory modular and distributed modes of face processing.

## Abstract (web summary)

Neuronal populations in the temporal cortex fire show increased activity in response to face stimuli. Here, the authors show using human intracranial recordings that face perception involves anatomically discrete but temporally distributed response profiles in the human ventral temporal cortex.

## Introduction

Studies using lesion methods^1,2^, functional imaging tools^3–6^, or scalp encephalography (EEG)^7^ and magnetoencephalography^8,9^ have offered invaluable causal, spatial, and temporal information about the neural mechanisms of face processing in the human brain. Work in non-human primates^10–15^ has also provided important and novel insights. However, despite great progress, the long-lasting controversy between modular versus distributed models of face processing has persisted in the literature^16^. Some studies have revealed face-selective responses in anatomically discrete regions of the temporal cortex (TC)^5^, and other observations have shown that the pattern of responses to face stimuli can be discerned from sampled data from non-selective regions of the TC^17^, suggesting that face information is anatomically distributed. Both theories have unfortunately relied on the information with limited temporal resolution averaged over multiple seconds or from methods using regions of interest and averaging across subjects, or direct recordings from a single or a pair of recording sites. Thus, the fast temporal dynamics of face processing across a large extent of the cerebral cortex within individual brains remains poorly explored.

Intracranial recordings in neurosurgical subjects with a large number of electrodes spread over a relatively large extend of the cortical surface—a method known as electrocorticography (ECoG)^18^—offers a new opportunity for acquiring fast temporal information from precisely localizable sources of signal. This method offers millisecond temporal resolution and millimeter anatomical precision in the subject’s own native brain space. Unlike the uniform spatial coverage of imaging methods, intracranial EEG (iEEG) relies on sampling from a limited number of implanted areas and leaves behind regions outside the coverage zones. While this leads to limited anatomical sampling, it may provide sufficient coverage for recording simultaneously from many sites within each individual brain, in order to explore the spatiotemporal dynamics of activity across different cortical areas. In addition, the intracranial approach allows delivering electrical pulses to discrete neuronal populations while causal changes in the subjective experience of the participant can be probed.

Using simultaneous recording across a relatively large area of the human brain one could test the hypothesis that face information is first processed within the most face-selective sites that are anatomically discrete and localizable within individual brains, which then is distributed to less selective sites.

While recent intracranial recordings and stimulation studies^19–24^, including our own^25–28^, have addressed the neurophysiological underpinnings of face processing in the human brain, to our knowledge, these studies have yet to address the notion of anatomical selectivity and temporal distribution of face information. The current study was designed to rely on a multiprong approach using univariate and multivariate methods of recording, as well as causal probing with electrical stimulation to test our proposed hypothesis.

We would like to emphasize here that the aim of the study was not to decipher the complex computational code of face perception in the human brain. Specifically, the study was not designed to address the nature of face processing in face-selective or non-selective sites. Studies in primate brains are perhaps better suited for that purpose. The goal of our work was twofold: (1) to explore the timing of response across different regions of the TC and test if there is distribution of face information in time, and (2) whether the stimulation of face-responsive sites cause the same effect in conscious viewing of faces. Here and in the remainder of the text, we will use the term “site” as the cortical region underneath a given electrode (~2 mm diameter). We use the term “face-selective site” to imply that neuronal responses in that site are significantly higher to face stimuli than non-face stimuli. We use the term “task-active site” to refer to sites in which visual objects induce significant activations compared to baseline, but responses to faces are not significantly different than responses to non-face stimuli.

Simultaneous recordings over a relatively large mantle of the human TC allowed us to address our two overarching questions. Here, we report that about half of recording sites show non-selective responses to at least one category of stimuli and only 10% of the sites have selective activations to face stimuli. Responses to face stimuli in the “task-active sites” are weak and correlate to the face responses present in face-selective sites. Both face-selective and task-active sites display a posterior to anterior gradient of selectivity as well as timing, suggesting that face information is distributed anatomically and in time. Finally, we report that only the stimulation of the posterior fusiform site (pFUS) region of the fusiform face areas (FFAs) appears to affect face perception.

## Results

### Data and design

We recruited eight neurosurgical patients implanted with ECoG electrodes as part of their presurgical invasive evaluation for medication-resistant focal epilepsy. Subjects had unilateral electrode implantation in the right (five subjects) or left (three subjects) hemisphere (Supplementary Table 1). Electrodes across all subjects (*n* = 357) provided suitable coverage over the ventral and lateral TC.

We recorded from each of the 357 implanted electrodes with high temporal resolution (>1000 samples per second) while the subjects performed a visual task, in which they viewed images of faces (human, mammal, bird, and marine), and non-faces, including bodies without faces (same four categories), limbs (human), objects, and places. Randomly, a red hashtag sign appeared on the screen (without any other superimposing images) at which time the subjects were instructed to press a key.

### Encoding of face information in the TC

**Fig. 1:**
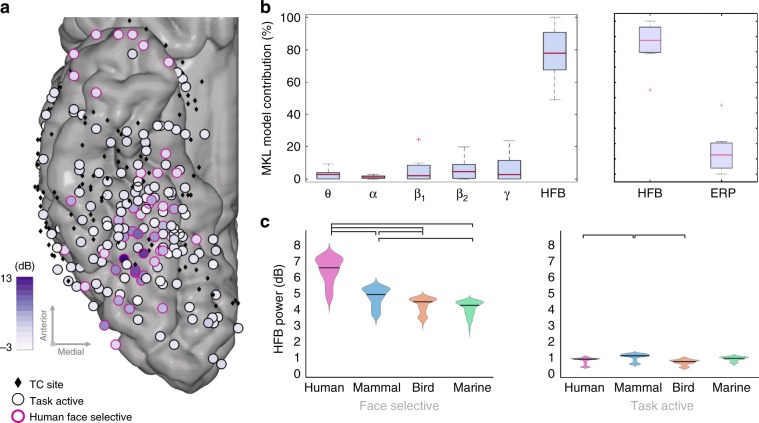
Effect of species on face coding. **a** Task-active and human face-selective sites across subjects in HFB. The coordinates of the electrodes from left hemisphere subjects were mirrored such that all sites could be displayed on a single hemisphere. Among the 357 included TC sites (represented by black diamonds), 190 were task active (represented by circles) as defined by permutation tests (i.e., presenting a significant response to at least one category). The difference between their response to human faces and non-face stimuli are displayed as a color-coded fill. In total, 48 task-active sites were identified as selective for human faces (represented with a pink contour). **b** HFB activity has the highest contribution in the discrimination between human faces and non-faces, within and across subjects. The results of the MKL model are plotted as box plots of the frequency band contributions to the model across the eight subjects, with the median represented by a red line. Similarly, HFB is favored over ERPs in an MKL model combining HFB power and ERPs. **c** HFB amplitude averaged within the [150 500]ms time window after onset for each of the four subcategories, in face-selective (left) and task-active sites (right). Coloring and initials represent the face subcategories: human (H/pink), mammal (M/blue), bird (B/orange), and marine (Ma/green). For face-selective sites, significant differences can be found (paired permutation tests, displayed by a black bracket) between the HFB responses to human faces and the mammal, bird, and marine faces, as well as between the mammal and the bird and marine faces. There is no significant difference between the responses to bird and marine faces. For task-active sites (i.e., active but not face selective), significant differences can be found between the bird faces and the human and mammal faces.

We quantified face information (i.e., the ability to discriminate faces from non-faces stimuli) in narrow bands of frequencies (*θ*: 4–7 Hz, *α*: 8–12 Hz, *β*_1_: 13–29 Hz, *β*_2_: 30–39 Hz, and *γ*: 40–69 Hz) as well as High-Frequency Broadband (HFB, 70–177 Hz) signals using univariate tests and a multiple kernel learning (MKL)^29^ method configured for iEEG signals^30^. Compared to the power of any other frequency band, the signal in the HFB showed the strongest univariate and multivariate changes in response to faces relative to non-faces (Fig. 1b, Supplementary Table 2). Similarly, the signal in the HFB was favored over evoked response potentials (ERPs) in a multivariate analysis. Given these findings, we focused further analyses on the HFB power and its profile of response across anatomical sites and task conditions.

We are mindful that the richness of the intracranial electrophysiological signal could have been explored by analyzing the power or phase of slower frequencies, or their coupling with higher frequencies^31^. However, the HFB signal is well suited for the purpose of testing our predictions not only because of our MKL findings, but also because of the large body of evidence from other human^32–37^ and non-human^38–43^ studies (as summarized in ref. ^18^) that have confirmed HFB power as a reliable correlate of hemodynamic signal and averaged single and multiunit activity of a population of neurons in a given cortical site. More importantly, HFB has a more precise anatomical source compared to lower frequencies^18^. The HFB signal hence provides a suitable marker for the engagement of a given cortical site in a given function. Similarly, the time of onset and the power of HFB provide valuable information about the time and level of engagement of a population of neurons adjacent to the recording electrode^41,42^.

Using HFB activity, we found a heterogeneous profile of responses across recordings sites (Supplementary Table 3). Of the recorded sites (*n* = 357), 53.22% (*n* = 190) had significant responses to at least one category of stimuli relative to baseline (i.e., “active” sites). A total of 13.45% recording sites (*n* = 48) showed selective activations to human faces compared to any other stimuli (“human face-selective” sites, Fig. 1a). Only 10.64% of the recording sites (*n* = 38) showed face-selective responses (comparing all subcategories of faces to all non-faces; i.e., “face-selective” sites*)*. The (human) face-selective sites were clustered in the fusiform gyrus or lateral occipital gyrus. While the group-level visualizations are presented in Montreal Neurological Institute (MNI) space^44^, for our anatomical claims, we rely on the precise location of the electrodes in each subject’s own brain space (Supplementary Figs. 1 and 2). Interestingly, while there was clear overlap between the “face-selective” and “human face-selective” sites, a few sites (17 out of 357 sites) showed selective responses to human faces while lacking significant responses to other face stimuli. In further analyses, we refer to the sites that were assessed as “active” and that were neither face nor human face selective as “task-active” sites.

To explore whether biological similarity of the face stimuli (humans and mammals versus birds or marine) influences the neural responses in the face-selective sites, we compared responses to faces of different categories. This analysis showed that human faces induced the strongest activations on face-selective sites (median = 5.21 dB, *n* = 1579) followed by mammal faces (median = 4.06 dB, *n* = 1609), bird faces (median = 3.48 dB, *n* = 1666), and marine faces (median = 3.48 dB, *n* = 1706, Fig. 1c left). All pairs of face subcategories showed a significant difference in HFB power (permutation test, *p* < 0.05 after Bonferroni correction), except for the comparison of bird and marine faces (human-mammal: *p* < 0.0001, human-bird: *p* < 0.0001, human-marine: *p* < 0.0001, mammal-bird: *p* = 0.0012, mammal-marine: *p* = 0 < 0.0001, bird-marine: *p* = 0.9896, permutation tests). In comparison, the amplitude of responses to subcategories of faces in the task-active sites showed lower HFB power for the face categories (human faces = 0.73 dB, *n* = 5761, mammal faces = 0.91 dB, *n* = 5497, bird faces = 0.67 dB, *n* = 5837, marine faces = 0.79 dB, *n* = 5808, permutation tests, Fig. 1c right). Only mammal faces elicited significantly higher responses than did human and bird faces (mammal–human: *p* < 0.001, mammal–bird: *p* < 0.0001). Other pairwise comparisons did not show differences in terms of HFB amplitude across the four subcategories of faces (permutation test, *p* > 0.05 after false discovery rate (FDR) correction). Please note that these univariate results were not driven by physical differences in the stimuli (Supplementary Table 4, Supplementary Discussion 4).

### Decoding of face information in the TC

Given that the HFB responses to human faces had the highest signal-to-noise ratio (SNR), further analyses focused on the processing of human face stimuli. To address the earlier imaging reports of distributed face processing^17^, we investigated the decoding of face information across human face-selective and task-active (i.e., non-selective) sites. In particular, we investigated whether task-active sites included face information that was not correlated with the face information present on selective sites. Such information could be too weak to be detected by our (stringent) univariate test or have another pattern than the one we assume in our test (e.g., a sinusoidal variation of the HFB signal for faces could lead to a mean of 0 dB over the considered time window). Instead of relying solely on decoding performance, we referred to a recently developed framework^45^ to infer causal relationships between the stimuli and the observed EEG activity in decoding (here machine learning-based modeling) settings. This approach allows to isolate the causal contribution of task-active sites following the presentation of face stimuli in face processing. Please note that in this analysis, the term “causal” relates to the relationship between the presentation of a stimulus (cause) and subsequent changes in brain activity (effect), and as such may not imply causality in terms of lesion studies or electrical stimulation experiments. The considered framework assesses the “relevance” of both face and task-active sites in discriminating between human face and non-face epochs instead of referring to decoding performance. A feature is assessed as “relevant” in decoding settings if, when removed, the performance of the model is significantly affected. This is similar to the procedure used in a recent publication^46^, in which the face information shared across regions of interest was taken into account. Features assessed as (respectively not) relevant in both encoding settings (corresponding to our univariate tests) and decoding settings are (respectively not directly) causally related to the stimuli.

**Fig. 2:**
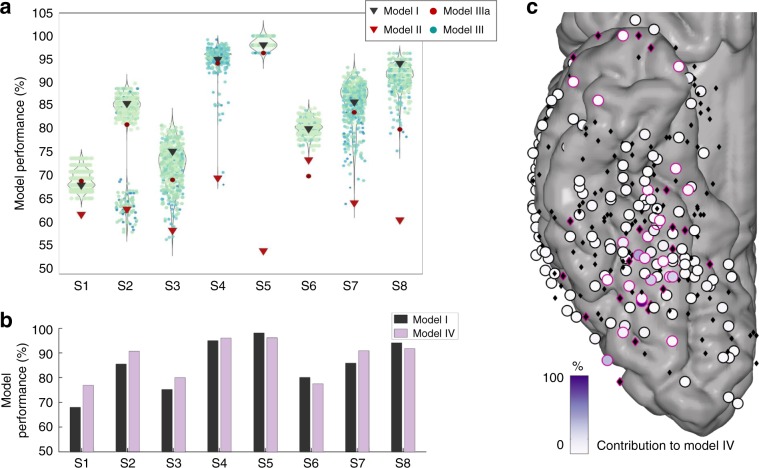
Nature and amount of face information on TC sites in decoding settings. **a** Dark grey triangles represent the performance of model I, i.e., including all TC sites. Performance of model II is represented by red triangles. Red circles represent the performance of model IIIa. Each colored dot represents one of the “random set” models (499 models per subject, model III), their color representing the proportion of face sites were included in the model (dark blue is one face site, light green is all face sites). Violin plots represent the distribution of the “random set” model performances compared to models I and II. **b** Sparse models perform as well as or better than distributed models. Bar plot representing the balanced accuracy for model I compared to the model accuracy of the sparse model IV, for each subject. **c** Site contributions to the sparse model, plotted across subjects. The contribution of the site (in %) is represented by a color-coded fill. Black diamonds have a perfectly null contribution to the model while circles had a positive contribution to the model. Sites assessed as human face selective by the univariate analysis are highlighted by a pink rim.

To address face processing in face-selective versus task-active sites, we ran three machine learning models discriminating between human face and non-face (i.e., human, mammal, bird, and marine body parts, places, objects, and human limbs pooled) stimuli, using signals from different sets of sites. The results from all models are displayed in Supplementary Table 5 and Fig. 2a. For each subject, model I incorporated data from all TC sites. For all subjects, model I was able to significantly discriminate between human faces and non-faces (permutation test *p* < 0.05, FDR corrected). Model II used the same classification but excluding the face-selective sites. Significant discrimination between faces and non-faces was only observed in two subjects out of eight (permutation test, *p* < 0.05 FDR corrected). In these two subjects, the significant decoding accuracy suggests that at least one site contains information about the discrimination at hand. This could however be related to selective responses to non-face stimuli, as we had not excluded sites selective to one or more subcategories of non-face stimuli. This first result suggests that face information on task-active sites is weak in the considered time window. Comparing models I and II revealed that there is a significant decrease in accuracy when excluding face-selective sites from the classification across subjects (Wilcoxon signed-rank test, *p* = 0.0078, *n* = 8). This result suggests that face sites are relevant in decoding settings for face processing^45^ and hence causally related to face stimuli. To test whether random subsets of task-active sites are relevant in decoding settings, we removed all task-active sites from the classification model (model IIIa). This led to an average decrease in model performance of 4.9% (Supplementary Table 5, Fig. 2a), with a large variability across subjects (min: +0.86%, max: −11.66%). While these results display a significant effect of task-active sites on model performance (Wilcoxon signed-rank test, *p* = 0.0234, *n* = 8), they were built using only ~13.45% of the features. To disentangle a decrease in feature space from a decrease in (face) signal, we built 499 models that included the same number of sites as model II by randomly selecting task-active and face-selective sites, but including at least one face-selective site. This means that across the 499 models (referred to as “random set” models), the proportion of face-selective sites varied, but was non-null. This procedure guarantees the same number of features as in model II and asserts whether removing random subsets of task-active sites affects performance. Across subjects, removing random sets of task-active and face-selective sites did not affect the model performance significantly (Wilcoxon signed-rank, *p* = 0.5781, Fig. 2a), nor did it within subjects for seven out of eight (Supplementary Table 5). For subject 2, a multimodal distribution is observed reflecting whether a specific face-selective site is included in the model (leading to accuracies >79%) or not (accuracy <70%, 83 models out of 499). There is a significant difference in model performance when this site is included or excluded from the model (*p* < 0.0001). The results from models IIIa and III suggest that task-active sites had a limited effect on the decoding model and were at best weakly relevant. Notably, we found a significant relationship between the proportion of face sites included in the analysis (i.e., from one face site to all sites, randomly selected in the 499 models) and the model performance for six out of seven subjects (Supplementary Table 5, *n* = 499). This result could arise from two scenarios (or a combination of the two): either face information on face sites is not redundant, i.e., each face site brings unique face information and/or including more sites leads to better SNR of the face pattern. In both cases, this result suggests that the pattern can be more easily identified when more human face sites are included.

The degree of inter-subject variability (both for model II and the “random set” model III) could not be explained by the number of trials (*ρ*(model II—#trials) = 0.5073, *p* = 0.1994, *n* = 8; *ρ*(median random sets—#trials) = 0.0068, *p* = 0.9872, *n* = 8) or of sites included in the analysis (ρ(model II—#sites) = −0.1221, *p* = 0.7734, *n* = 8; ρ(median random sets—#sites) = 0.00156, *p* = 0.9708, *n* = 8). The inter-subject variability, however, could be explained by various factors, such as SNR, amount of correlated noise or placement of the electrodes, which are complex to quantify. Please note that due to the limited numbers of trials per subject, we were not able to perform the univariate and multivariate analyses on different partitions of the data. See additional analyses in Supplementary Table 6 and Supplementary Discussion 6.

To further investigate face processing in task-active sites, we performed the same classification as for model I, except that the considered algorithm enforces sparsity at the site level, i.e., it automatically selects a subset of sites to perform the classification. Comparing sparse and non-sparse modeling techniques is a common machine learning strategy to investigate data properties: Support Vector Machine (SVM) (i.e., model I) assumes that the information is fully distributed across features, by construction. If this assumption is correct, it should perform better than a sparse model. In contrast, if the sparse model performs better than or as well as model I while including only a subset of sites, it suggests that the information contained in the selected subset of sites is sufficient to perform the classification and that the non-selected features do not bring further relevant information. As in previous publications^30,47^, we used the simple MKL algorithm^29^ to perform the sparse modeling. Please note that the basis algorithm for the simple MKL is an SVM, hence the effect of implementation on the results is limited^29^. The results are displayed in Fig. 2b and Supplementary Table 7. Across all subjects, the sparse model performs slightly better than the distributed SVM model (Wilcoxon signed-rank test, *p* = 0.25, *n* = 8, average increase in performance of 2.28%). In five subjects out of eight, there is a clear increase in performance, of up to 8.95% for subject S1. In subjects 5, 6, and 8, the difference in model performance between model I (SVM) and model IV (sparse MKL) is around −2%. Of note, in two subjects the accuracy of the model was over 90%, which left little room for improvement (S5 and S8). This accuracy was reached with 35% of the sites being included, displaying that most sites did not contribute to the model. We however preclude from relating a contribution to the model to face processing as some sites can act as “noise canceller”^48^. In addition, the sparse model weight maps significantly overlapped with the univariate maps of human face selectivity across subjects (*ρ* = 0.4708, *p* < 0.0001, Fig. 2c) and within subjects for seven out of eight subjects (except subject 8, Supplementary Table 7, Supplementary Fig. 2). This result shows that MKL relies heavily on face-selective sites for the classification. For subject 8, the classifier seems to rely on non-face information, as the site with highest contribution contains higher amplitude signals for non-face categories than for human faces (pooled non-face > face: *p* < 0.05, permutation test). These results suggest that the potential face information present in task-active sites is correlated with the information present in face-selective sites. Please note that this analysis does not suffer from circularity as it does not rely on the previous univariate results.

### Temporal distribution of face processing

Thus far, our univariate and multivariate results display that face sites are “relevant” for human face processing. However, we also showed that including more face sites in the model increases model performance (random set models). We then explored the relationship between selectivity, anatomy, and timing of the HFB responses on face and task-active sites during human face processing.

While the face sites displayed significant responses to human face stimuli, the selectivity of their responses decreased from posterior to anterior sites as demonstrated by correlating the MNI *y*-coordinate with selectivity (*ρ* = −0.6097, *p* = 5.39e−06, *n* = 47). For task-active sites, selectivity to human faces increased from posterior to anterior sites (*ρ* = 0.2699, *p* = 0.0013, *n* = 139).

**Fig. 3:**
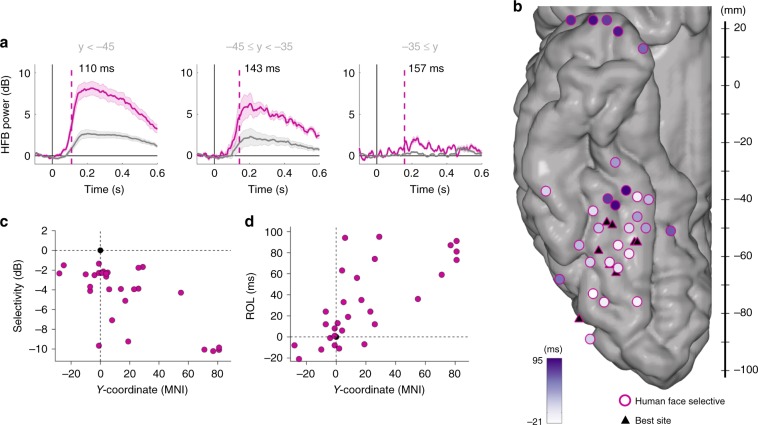
Temporal distribution of human face information on face-selective sites. **a** Average traces and ROL according to *y*-coordinate. Mean (±standard error) HFB traces across face-selective sites in the posterior (<−45, *n* = 24), middle (−45 to −35, *n* = 7), and anterior (after −35, *n* = 6) TC sites for human faces (pink) and non-faces (grey) stimuli. **b** Response onset latency (ROL) over human face-selective sites for the human face category, as represented by a purple color scale fill on the MNI cortex with the best (i.e., most human face selective) site in each individual chosen as the point of reference (triangles). Here only relevant electrodes are shown. **c** ROL in each subject, when compared to MNI *y*-coordinate with the best site in each individual chosen as the point of reference. Latency is related to anatomical location of the sites. **d)** Selectivity in each subject, when compared to MNI *y*-coordinate with the best site (i.e., most human face selective) in each individual chosen as the point of reference. The best site is represented as a black circle, at the crossing of the two axes. Selectivity is related to anatomical position.

**Fig. 4:**
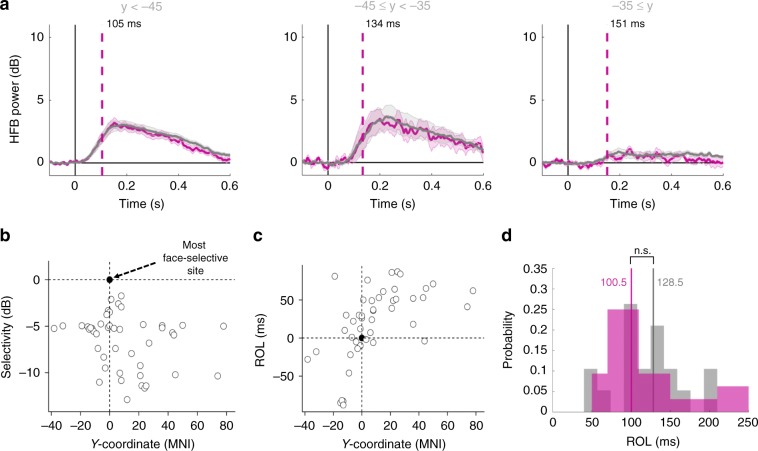
Temporal distribution of human face information on task-active sites. **a** Mean (±standard error) HFB traces across task-active sites in the posterior (<−45, *n* = 24), middle (−45 to −35, *n* = 7), and anterior (after −35, *n* = 6) sites for human faces (pink) and non-faces (grey) stimuli. **b** Selectivity in each subject, when compared to MNI *y*-coordinate with the best (i.e., most human face selective) site in each individual chosen as the point of reference. The best site is represented as a black circle, at the crossing of the two axes. Selectivity is not related to anatomical position. **c** ROL in each subject, when compared to MNI *y*-coordinate with the best site in each individual chosen as the point of reference. Latency is related to anatomical location of the sites. **d** Histograms (in probabilities) of ROL values for human face-selective sites (*n* = 32) and their matched task-active sites in terms of *y*-coordinate (*n* = 19). N.s. refers to a non-significant difference between the two distributions.

In addition, our fast event-related paradigm combined with simultaneous recordings across selective and task-active sites with high sampling rate allowed us to compare the latency of neuronal population responses to faces within the first 500 ms of stimulus presentation. We measured the response onset latency (ROL) to human face stimuli across human face-selective sites and task-active sites based on unsmoothed, normalized signals (Figs. 3, 4). A recent study^49^ revealed a temporal lag between the posterior fusiform gyrus (pFG, *y* < −45) and medial fusiform gyrus (mFG, −45 < *y* < −35) across face-selective sites. In line with these anatomical boundaries, our data indicates an average temporal delay of 33 ms across the two regions and a further delay of 13 ms with the more anterior face-selective region (*y* > −35). Furthermore, ROL values across human face-selective sites were significantly correlated with the *y*-coordinate of the corresponding site (*ρ* = 0.5207, *p* = 9.5399e-04, *n* = 37). Interestingly, the posterior-to-anterior lag was also significant for task-active sites (*ρ* = 0.6563, *p* = 7.3554e−07, *n* = 46, Fig. 4). Importantly, the group-level findings presented here were also present at the individual subject level for human face-selective sites, when calculating the ROL for each site relative to the most human face-selective site within subject (Fig. 3b–d): *ρ*(ROL−*y*) = 0.7593, *p* = 1.7911e−06, *n* = 29 and ρ(selectivity−*y*) = −0.5680, *p* = 0.0013, *n* = 29. In other words, the posterior to anterior ROL gradient was not driven by one or a few subjects who happened to have coverage over a specific region of the TC. For task-active sites (Fig. 4b, c), the correlation between ROL and y-coordinate was also significant: *ρ* = 0.6718, *p* = 3.2032e-07, *n* = 46. On the other hand, there was no significant correlation between anatomical position and selectivity when compared to the most face-selective site: *ρ* = -0.2269, *p* = 0.1294, *n* = 46. We remind the reader that task-active sites showing non-selective responses can be of two types: those in the earlier visual cortices (located more posterior than the first face-selective sites) and engaged much earlier in presumably low-level processing of visual information and those located in the anterior temporal lobe that are engaged much later in presumably high-level processing of visual information.

We explored the timing of events across task-active and face-selective sites (both including posterior and anterior sites), and compared the ROL values between the two groups, but this comparison was not significant (*p* = 0.9452, Wilcoxon rank sum test). We argued that the lack of difference could be due to large variability in ROL values within each group. To mitigate this confounding effect, we matched face-selective sites to their closest task-active sites in terms of *y*-coordinate (up to 2 mm difference), and found that the time of activation in face-selective sites trended earlier than anatomically matched adjacent non-selective sites but this difference did not reach statistical significance (paired Wilcoxon signed-rank, *p* = 0.1631, *n* = 32 face sites, and *n* = 19 task-active sites). Performing the matching within subjects led to similar results (*p* = 0.1752, *n* = 20 face sites matched to 16 unique task-active sites). Figure 4d shows the distribution of ROL values across the two groups after matching.

Importantly, signal amplitude or slope did not affect our ROL method (Supplementary Fig. 4, Supplementary Discussion 8).

### Effect of electrical brain stimulation on face perception

Lastly, we explored the effect of electrical perturbation of face-selective sites on the subjective processing of faces. We hypothesized that the stimulation of face-selective sites in the posterior TC would cause more salient effects than the stimulation of non-selective sites in the anterior TC. This hypothesis has not been addressed in prior to electrical stimulation studies showing distortion in conscious viewing of faces^26–28,50,51^ or naming of famous faces^52,53^. Subjects were instructed to view a human face at the bedside while we performed active or sham (zero current) stimulations of selective, task-active, or non-responsive sites. Subjects then reported whether the face remained the same or was distorted. We emphasize that this anecdotal report departs from the well-controlled experimental procedures that have been performed in non-human primates and human subjects due to limitations in our equipment and clinical time constraints.

**Fig. 5:**
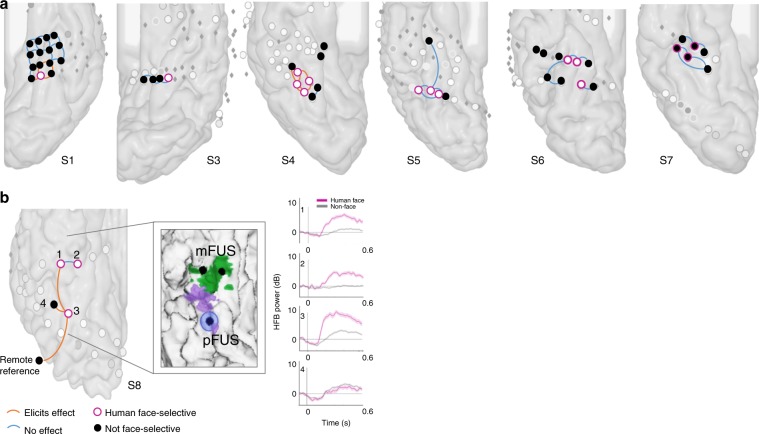
Electrical brain stimulation. Effect of electrical stimulation applied to a pair of electrodes is shown with lines colored in orange (stimulation of this pair affects subjective face perception) or blue (stimulation of this pair has no effect on subjective face perception). Human face-selective electrodes are outlined in pink. **a** Depicts the localization of stimulated sites on subjects 1, 3, 4, 5, 6, and 7 using the same convention as in Supplementary Figs. 1 and 2. **b** Displays the same results for S8 and investigates further the localization of the stimulated sites. In the inset figure, the location of sites 1, 2, and 3 are shown on top of the fMRI patches of mFUS (green) and pFUS (purple). The estimated cortical area affected by the stimulation of site 3 is shown with a blue circle around it. The cortical area was estimated using the parameters recently validated in a separate cohort^54^. Site 3 in subject 8 is the site whose stimulation caused distortion of faces when it was stimulated in pairs with site 1 (another face elective site), nearby site 4 (task-active site), and a remote reference site. Right panel shows the HFB responses to human faces (pink) and non-faces (grey) of the sites that were stimulated. Please see Supplementary Movie 1 for the video of subject S8’s verbal responses after being stimulated.

Figure 5 summarizes the results of electrical stimulations and details of the procedure in each patient is provided in Supplementary Table 9. Our findings indicate that distortion of human face perception was reported only with real (but not sham) stimulation of some (but not all) face-selective sites. Among 52 stimulated sites in the fusiform area, 24 were face selective and 28 were not. Among the 24 face-selective sites (17 pFG sites and 7 mFG sites), 6 caused distortions in face perception when stimulated while 18 did not; all 6 sites that caused distortions in face perception when stimulated were located in the posterior fusiform gyrus (*y* < −45). Among the 28 non-selective sites (14 pFG sites and 14 mFG sites), 3 caused distortions in face perception when stimulated while 25 did not; again, all 3 sites that caused distortions in face perception when stimulated were located in the pFG. We found a significant effect of anatomical site in predicting the subjective responses to electrical stimulation: one-tailed *z-*score tests for two population proportions showed that the proportion of sites whose electrical stimulation induced a face distortion in the pFG (9/31) versus the same proportion in the mFG area (0/21) was significantly different (*z* = 1.833, *p* = 0.034). This trend held true when comparing the proportion of pFG versus mFG sites that distort face perception when stimulated within face-selective sites only (pFG: 6/17; mFG: 0/7; *z* = 1.815, *p* = 0.035) and within non-selective sites only (pFG: 3/14; mFG: 0/14; *z* = 1.833, *p* = 0.034). These results indicate that electrodes that cause distortions in face perception when stimulated are greater in proportion in the pFG than in mFG.

Moreover, among the nine pFUS sites that caused face-perception distortion, six were face-selective sites and three were sites immediately abutting the face-selective sites within the likely area of distribution of electrical charge. In fact, two of the abutting task-active sites displayed weak face selectivity (permutation test, *p* < 0.05 before FDR correction) during face processing. Based on our previous quantification of the spread of electrical charge in the human brain^54^, we believe that the electrical fields generated by the stimulation of the three task-active sites likely spread to the abutting face-selective sites.

Of note, face distortion occurred only with right hemisphere stimulations except in one left hemisphere case (S1) who happened to be left handed. We have previously discussed the lateralized effect of stimulation in a separate report^55^. As such, we find that stimulation of posterior face patches, in the language non-dominant hemisphere, causes face specific distortions.

In one subject (subject 8), we probed the effect of stimulations across two patches of the FFA (sites numbered as 1, 2, and 3 in Fig. 5b). Using bipolar (adjacent pair of sites stimulated together) or unipolar stimulations (site was paired with a distant reference), we elicited distortions when the subject looked at his own face in the mirror, looked at a cartoon face drawn on a piece of paper, and focused on parts of a face (e.g., eyes or lips). He also reported induced perceptions of a face during electrical stimulation while his eyes were closed. The subject’s verbal reports after stimulation include “one side of the face changed”; “facial features [turned] into a cartoon”; one eye “became someone else’s”; “face wiggled a little bit”; and “face looked familiar” (Supplementary Movie 1). More importantly, the effects were observed only when site 3 was stimulated. To explore the anatomical location of site 3, we localized the pFUS and the medial fusiform face area (mFUS) onto subject 8’s native neuroanatomical space. Using methods described in ref. ^56^ the calculated field of electrical stimulation of site 3 was precisely localized in the pFUS (Fig. 5b, zoom panel). The other face-selective sites (1 and 2), located in mFUS failed to cause any distortions even though they were only 1 cm away from site 3. This finding adds to our previous report^28^ in which we stimulated both mFUS and pFUS in a bipolar manner.

## Discussion

Our study addresses the spatiotemporal distribution of face information based on univariate measures, machine learning-based modeling, timing analysis, and direct cortical stimulation. Using this multipronged approach and by leveraging the temporal resolution of the ECoG method, we confirmed the following results: the majority of recording sites in the human TC do not show any significant change of activity in response to the visual presentation of faces; a minority of sites respond non-selectively to at least one category of visual stimuli; and only fewer sites respond selectively to face stimuli, and that these are clustered in the fusiform gyrus or lateral occipital gyrus across individuals.

We found that face information was represented to a greater degree in face-selective regions than in task-active sites. Removing face-selective sites from machine learning-based classification significantly dropped the decoding accuracy of the model. In addition, classification model suggested that task-active sites had a limited effect on the decoding model and were at best weakly relevant (in the technical sense as used in Weichwald et al.^45^). This was supported by an improvement in model performance when considering a sparse approach. Overall, our results suggest that face information in task-active sites is weak, highly correlated with the face information that is present (earlier and stronger) in face-selective sites, and not causally necessary nor sufficient (in terms of our machine learning analysis) for face processing. We confirm the presence of temporally distributed information within task-active and face-selective sites that may be functionally relevant and even necessary for downstream processing of faces (e.g., associating faces with memory or name information).

Comparing our results to prior neuroimaging studies, we would like to emphasize the methodological differences including the number of recording sites, the time scales considered, and the recorded signals used in the analysis. For instance, the number of anatomical samples in each subject is limited with the ECoG method, which can reduce the power of the algorithm in detecting weak, distributed patterns. To maximize the detection of small effects, we used Support Vector Machine classifiers, which are known for their ability to detect subtle, distributed patterns and we focused our analysis on the high SNR of HFB responses induced by human face versus non-face stimuli. While we could not relate the number of sites to model performance (Results section), it is possible that the signal in task-active sites is too weak to be detected across a few tens of anatomical samples in each subject. On the other hand, the novelty of our data is in part due to the high temporal resolution of ECoG, which enabled us to measure the fast temporal dynamics of face processing in the human brain. Specifically, our electrophysiological analysis relied on responses elicited within 500 ms of stimuli presented for 300 ms with an inter-stimulus interval of 400 ms. This is in stark contrast to some of the classic neuroimaging studies whose temporal window included >10 s of signal processing (e.g., 24 or 16 s long blocks of visual stimuli for each category^17^).

Another methodological caveat that needs to be considered is that the task-active areas in humans may be variable in their relative size and primary cytoarchitectonic composition^57^. Therefore, hubs of activity in posterior to anterior TC may have different sizes or shapes of physiological responses or locations on the surface of gyri versus depth of sulci which could have influenced the properties of recorded electrophysiological signals as well as the subjective effect of electrical stimulation. As such, the results reported here could represent an idiosyncrasy of our iEEG method. However, it is still noteworthy that responses to faces were significantly faster in the posterior sites than in anterior face-selective sites. By using ECoG recordings, our results confirm our overarching hypothesis that face information is anatomically localized but temporally distributed. The posterior to anterior gradient was observed within both face-selective and task-active sites, with no statistically significant time differences between task-active and face-selective sites—though the time of activations in face-selective sites trended earlier (Fig. 4d). The lack of statistical significance in terms of ROL can be explained by several factors. First, there is a posterior-to-anterior temporal lag in activation, i.e., anterior face-selective sites may respond later than posterior non-selective task-active sites. Second, there are task-active sites in the more posterior regions that respond significantly earlier than any other sites. For instance, one cannot assume that face-selective activations precede the ones in the primary visual cortex. Third, given these two confounds, the number of electrodes in each subject may be too small for statistically differentiating a subtle temporal lag between selective and adjacent non-selective sites. Finally, despite the trending results reported here, we would caution against any conclusions from it. Given our knowledge of brain anatomy, we would anticipate that task-active and face-selective sites would be engaged in bilateral information exchange at multiple levels along the posterior—anterior axis of signal propagation and, as such, the ROL values across sparse recording sites in each individual brain may not be able to address this issue and addressing it with iEEG may be ill-advised to begin with.

Additionally, our findings support the causal link between some, but not all, face-selective sites of the fusiform gyrus and conscious processing of faces as suggested previously by our group^26–28^ and others^51,52^. Our findings from electrical stimulation procedures (1) replicate our prior findings^26,28,55^ in a new cohort of subjects that the stimulation of the right (non-language dominant) hemisphere is more important for eliciting subjective reports of face distortion; (2) that some of the posterior face-selective sites (i.e., *y* < −45 in MNI space) are more critical than anterior sites for conscious processing of faces, and (3) that the pFUS (but not the mFUS) region may serve as a key node in the conscious processing of faces. While the low numbers of anterior site stimulations preclude any definite conclusions, the third finding is intriguing and needs to be replicated in a larger sample of subjects with a task-based and stimulus-locked electrical perturbation procedures. As demonstrated in non-human primates^14,58^, and also in the human lateral occipital and fusiform cortices^19,22,56^, we acknowledge that the face-selective sites outside the fusiform gyrus also play an important role in face processing. For example, a recent study in non-human primates showed that the micro-stimulation of the anterior face-selective patch (area anterior medial) severely distorted the monkey’s percept of facial identity, such that faces depicting the same identity appeared to depict different identities^58^.

Our findings may provide neurophysiological explanations for some of the classic behavioral observations. The preferential responses to human faces compared to non-human faces is in line with a recent human functional magnetic resonance imaging (fMRI) study, in which multivariate patterns of activity throughout the ventral TC were found to correlate with behavioral judgments of biological similarity of the same stimuli (i.e., monkeys and mammals versus insects and birds)^59^. Moreover, two electrophysiological studies in the macaque brain have shown faster responses of neurons to primate faces compared to non-primate animal faces^60^ or a change in species selectivity across different patches of face-selective areas^61^. Our findings along with these studies may explain the behavioral observations of conspecific advantage in face recognition; that is, humans and other primates recognize members of their own species more readily than faces of other species^62,63^. For instance, chimpanzees raised in a human environment are better at discriminating pictures of unknown human faces than unknown chimp faces^64^.

In closing, our data suggest that a few anatomically discrete sites play a crucial role in processing face information and that there is a time delay in their processing of the same information. However, our study does not suggest that the conscious perception of faces solely depends on the operation of these isolated patches of face-selective sites. We acknowledge that different facets of face processing may occur in different patches of face-responsive sites and that these neuronal patches are embedded in a larger network of visual and other association areas of the brain, and their function should not be seen in isolation from this wider integrated brain network^16,65^. Future studies with simultaneous recording across face-selective and other association areas are needed to determine how different facets of face information is encoded in each of the face patches and how their function is embedded and relayed to the rest of the brain for serving human cognition and behavior.

## Methods

### Demographics and recordings

Eight subjects (six males, two females, aged between 23 and 68 years) were implanted with intracranial electrodes to localize the source of drug-resistant seizures. The procedure was approved by the Stanford Institutional Review Board and the subjects provided written informed consent to participate in the study. The location of the grids was determined by clinical needs (Supplementary Fig. 1, three left hemisphere implantations, five right). Data were obtained at 1525.88 Hz through a 128-channel recording system (Tucker Davis Technologies) for the first seven subjects while a Nihon Kohden Technology system with simultaneous video monitoring was used to perform 1 kHz recordings in subject S8. Each electrode was a platinum plate, either 2.3 mm or 1.15 mm in diameter (exposed recording area) with center-to-center spacing of 4–10 mm between adjacent electrodes on the grid or strip. Electrodes containing artifacts or pathological activity were discarded from further analyses.

### Anatomical localization of electrodes

Structural MRIs were acquired with a GE 3-Tesla Sigma scanner at Stanford University equipped with a head coil of a T1-weighted SPGR pulse sequence. The images were AC–PC (anterior commissure–posterior commissure) aligned and were resampled to 1 mm isotopic voxels, then segmented to separate gray and white matter. Postimplantation computed tomography images were aligned to the pre-op MRI anatomical brain volume^66^. Electrodes were visualized on the subject’s own brain volume and reconstructed onto a three-dimensional cortical surface allowing for accurate anatomical localization of electrodes. The electrode positions were also transposed into the MNI space and displayed on a MNI cortex file for visualization of results across subjects. The coordinates of the left hemisphere sites were mirrored such that all sites could be displayed on a single hemisphere.

### Experimental paradigm

The experiment was administered using psychtoolbox running on Mac OSX. The laptop was placed ~70 cm from the subject’s eyes at chest level. Screen resolution was 1280 × 800. Each image was subtended five visual degrees at its longest dimension. Each subject underwent a visual task during which images of different categories were presented at the center of the screen for 300 ms, with an ISI of 400 ms. The categories included ten human face, human body, mammal face (one monkey face), mammal body, bird face, bird body, marine face, marine body, human limbs, object, and place. The image backgrounds were phase scrambled at 3% in order to reduce visual artifact. The visual dimensions of the image plus its scrambled background were 11.10 × 11.10 cm, and the visual angle was 9 degrees. Each category comprised 25 images, presented twice. This hence leads to 50 stimuli per category and 550 visual stimuli in total. During image presentation, the subject was asked to press a key (“press 1”) when the pattern “###” appeared in red at the center of the screen (further referred to as a “Response Block”). The onset of each stimulus was recorded by a photodiode signal generated by a luminance change in the display at image onset.

### Signal preprocessing

All preprocessing steps were performed using Matlab (The MathWorks, Inc., Natick, Massachusetts) and the SPM (www.fil.ion.ucl.ac.uk/spm) toolbox in custom routines (https://github.com/LBCN-Stanford/Preprocessing_pipeline). The data were first down sampled to 1000 Hz and filtered for power-line noise (band-stop between 57 and 63 Hz) and harmonics (117–123 Hz and 177–183 Hz). Sites underwent an automatic quality assessment: sites with variances five times larger or smaller than the average variance across all sites were labeled as pathological and excluded. Sites with three times more “jumps” (defined as changes in the signal derivative >100 μV) than the average across sites were considered as spiky and excluded. The signal was then re-referenced to the average of the signal over all selected sites. Each event was extracted (i.e., epoched) in the −200 to 700 ms time window around its onset and baseline correction was performed (using the [−200 to 0]ms time window around onset as baseline). Events were marked as artifacts if they contained spikes of >100 μV and were discarded from further analyses. At this stage, there were no further checks for interictal activity. A time-frequency decomposition was then computed using a seven-cycle Morlet wavelet, with frequencies ranging from 70 to 177 Hz (steps of 1 Hz, avoiding discarded frequencies from Notch filtering). A similar five-cycle Morlet wavelet time-frequency decomposition was performed for frequencies ranging from 1 to 69 Hz. The power in each frequency and time bin was rescaled using the log-power of the [−100 0]ms window around stimulus onset. Six frequency bands were considered in this work: *θ* (4–7 Hz), *α* (8–12 Hz), *β*_1_ (13–29 Hz), *β*_2_ (30–39 Hz), *γ* (40–69 Hz), and HFB (70–177 Hz). The signal was finally averaged across frequency bins within each band considered and smoothed with a 50 ms width Gaussian window. The HFB power in the [−100 600]ms window around stimulus onset was considered for further analysis (see Supplementary Table 2 and Supplementary Discussion 2 for justification).

### Relevance of TC sites in encoding

For each subject, sites were defined as “active” if they displayed a significant HFB response in the [150 500]ms after stimulus onset for at least one category, when compared to the event baseline ([−100 0]ms before onset). Paired non-parametric permutation tests (50,000 permutations) assessed the significance of the response (*p* < 0.05, FDR corrected for the number of sites tested). Out of the active sites, “face-selective” sites were identified as sites where significantly higher responses were seen to the four face categories (human, mammal, bird, and marine) pooled compared to all other stimuli pooled (non-parametric permutation tests, FDR corrected) in the time window [150 500]ms after onset. Finally, “human face-selective” sites displayed significantly higher responses to human faces compared to all non-face stimuli pooled (non-parametric permutation tests, FDR corrected) in the [150 500]ms after onset window. Active sites that were not assessed as face selective or human face selective are further referred to as “task-active” sites. Sites assessed as (human) face selective were considered as relevant in encoding settings for face processing^45^. These analyses were performed for the HFB signal but also for other frequency bands (Supplementary Table 2). The four face subcategories were then compared on both the “face-selective” and “task-active” sites based on the amplitude of their HFB response averaged in the [150 500]ms time window after onset. Permutation tests assessed the significance of potential differences between subcategories (10,000 permutations, FDR corrected for the number of tests (*n* = 12, 6 binary comparisons for face sites and 6 for task-active sites)). Please note that there is no circularity in this analysis as the contrast to select sites (i.e., faces versus non-faces) is different from the effect investigated (i.e., human faces versus mammal faces versus bird faces versus marine faces).

### Frequency information for faces

For each frequency band, a univariate analysis assessed active, face-selective, and human face-selective sites. In addition, a MKL^29^ model assessed the contribution of each frequency band to the discrimination between human faces and non-faces^30^. In this case, a linear kernel is built for each frequency band. Those kernels are then combined during the modeling step, based on a sparsity constraint. The model outputs a contribution for each kernel that can be interpreted as the weight of each frequency band in the classification. All modeling parameters were kept consistent with models I, II, and IV (see “Relevance of TC sites in decoding”). In addition, a second MKL model combined the power in HFB and the ERP (i.e., before time-frequency decomposition) as a recent study^67^ showed that ERPs could bring complementary decoding information to HFB signals. These analyses were performed in version 3.0 of the Pattern Recognition for Neuroimaging Toolbox (PRoNTo^30,68^).

### Low-level image features

To ensure that low-level features in the stimulus images did not drive our results, we performed multiple control analyses.

First, spatial frequency power spectrum with rotational average was calculated for each stimulus and averaged across categories. Averaged spatial frequency power spectrums were compared using the one-way Anova and post hoc analysis was performed using “Tukey–Kramer” method. Power spectral analysis returned 153 values for each image, which were averaged within each category (*n* = 153). We also computed the mean luminance across pixels in each category, pooling all non-faces together.

We then investigated the effect of mean luminance on the univariate results (i.e., on sites defined as face selective). To this end, we plotted the histogram of mean luminance in the face and in the non-face categories. We defined as low (resp. high) luminance faces, face stimuli with a mean luminance smaller (resp. larger) than 120 (threshold defined arbitrarily). The neural signals in each category was compared on all face sites (high luminance faces: *n* = 45, human:15, mammal:12, bird:7, and marine:11; low luminance faces: *n* = 55, human:10, mammal:13, bird:18, and marine:14), using permutation tests.

### Relevance of TC sites in decoding

We then assessed the relevance of face and task-active sets of sites in decoding settings. To this end, a machine learning model discriminating between human face epochs and non-face epochs was estimated based on different site sets: (I) All TC sites included for analysis (referred to as the “TC” model); (II) All TC sites, excluding the ones assessed as “human face selective” by the univariate, permutation tests (referred to as the “TC-sign” model); (IIIa) All TC sites excluding task-active sites; and (III) 499 random subsets of task-active and face sites, including at least on face site. In scheme (III), further referred to as the “random sets” model, the number of sites (i.e., features) included for modeling is identical as for model (II). However, the proportion of face sites randomly varies, from 1 to all face sites. The different models aim at answering the following questions: (I) Is it possible to significantly discriminate human face from non-face trials in each subject? (II) Are face-selective sites relevant for the discrimination between human faces and non-faces? i.e., do we observe a significant change in model performance when removing the set of face-selective sites? Accessorily, is it still possible to significantly discriminate between human face and non-face trials? Or, on the contrary, is the information in those human face-selective sites necessary for significant classification? (IIIa and III) Are (random subsets of) task-active sites relevant for the discrimination between human face and non-face stimuli? i.e., is model performance significantly affected when removing random (sets of) task-active sites? This analysis was conducted in PRoNTo version 3.0^30,68^. The data considered focused on the [150 500]ms after stimulus onset. The “mammal body”, “bird body”, “marine body”, “human body”, “object”, “place” and “limbs” categories were pooled together to form the “non-face” category. As this leads to imbalances in terms of the number of trials in each class (maximum 50 human faces compared to maximum 350 non-faces), epochs from the “non-face” category were randomly subsampled to closely match the number of epochs in the “human face” category. During this process, care was taken to include approximately the same number of epochs from each subcategory (e.g., 7 “mammal body”, 8 “bird body”, 7 “marine body”, 7 “human body”, 8 “object”, 7 “place”, and 7 “limbs”). A linear kernel matrix was built based on the data from all sites considered in the feature set (number of features = number of sites × 351 time points). This matrix corresponds to a similarity matrix between each pair of epochs (dot-product). The similarity matrix was then input into a Support Vector Machine classifier. It should be noted that SVM is an L2-norm regularized technique. This means that it does not assume or enforce a sparse distribution of the model weights. It should hence, in theory, be able to identify subtle, distributed patterns over the TC. Model performance was computed based on a five-folds cross-validation, i.e., 20% of the epochs were left out before training the model on the 80% remaining epochs (non-overlapping). The model was then tested on the left out 20% epochs and the predictions it returned were compared to the “true” targets. This partitioning of the data was performed five times in total, each partition corresponding to a “fold”. The model performance in this work was averaged across folds. To estimate model performance, the sensitivity for each class was computed (corresponding to the class accuracies for “faces” and “non-faces”). Those values were then averaged to provide a global measure of model performance, further referred to as “balanced accuracy”. Within each fold, another four-folds cross-validation was performed to optimize the soft-margin hyperparameter of the SVM model (*C* = 0.01, 0.1, 1, 10, or 100). The significance of the obtained model performance (*p* < 0.05) was assessed using 1000 permutations^69^. Classification accuracy was considered significant if the balanced accuracy was significant. In addition, the difference between each model and the “TC” model (I) was tested for significance based on Wilcoxon signed-rank test at the population level (*n* = 8). To ensure a fair comparison between those models, all modeling parameters were identical, including cross-validation folds, epochs considered and permutations of the labels. Only the sets of sites considered for modeling differed across the four schemes.

### Sparse versus distributed decoding information

Assessing relevance in encoding and decoding settings assumes the interpretation of negative results. As negative results are by nature inconclusive, we here investigate the effect of priors on model performance. For one model (model I), the algorithm used assumes a “distributed” prior (SVM, L2-regularization), i.e., all features contribute to the model. If this prior is appropriate, model performance should be high, and potentially higher than for other types of prior. We test this hypothesis by comparing model I to a model that enforces sparsity on the sites. Thereby, if the sparse model performs better or as well as a distributed model, the face information is likely localized in a few sites and other features bring no further relevant information. The sparse model, further referred to as model IV, is based on the sparse MKL method^30^. The main difference with model I is that sparsity is enforced at the site level. In practice, one linear kernel is built per site (i.e., number of features = 1 × 351 time points) and those kernels are combined through a sparse prior (L1-norm regularization). Some sites will hence not be considered in the final model (i.e., their contribution is zero). All modeling parameters are identical between models I and IV. Furthermore, the implementation of the sparse algorithm relies on an SVM (for each kernel), which limits the effect of implementation on the results. From the output of model IV, a “contribution” map can be built, which displays the contribution of each site to the final decoding model. Thereby, the “sparsity” of the model can be estimated as the number of sites with a non-null contribution to the model divided by the number of sites considered^70^. This measure was computed within subjects for each fold, then averaged across folds. Interpreting contribution maps is controversial^48^ as the amplitude of the contribution does not necessarily reflect the presence or absence of the signal of interest on a site. In this work, we correlate (Pearson correlation) the model contribution at each site with its human face selectivity to ensure that decoding is performed using information from face-selective patches, both within and across subjects. We however preclude from any conclusion relating contributions to the model and contributions to face processing.

### Circular analysis

In this work, we perform various analyses on the same data set, and more importantly, the contrasts investigated are identical in the univariate and multivariate analyses. The ideal solution would be to split our data set in two parts: one to identify face and human face-selective sites using univariate methods, the other to build the machine learning-based models. However, with numbers of trials as low as 30 for human faces after artifact rejection, splitting our data set would be detrimental for both analyses. In this section, we perform univariate analyses on another visual task recorded on the same subjects that includes human faces and non-face stimuli, to investigate the amount of task dependence on the identified sites. It is important to note that this visual task comprises a different contrast from our main task, as the visual task mostly comprises images of words and numbers, and not pictures. This explains why we chose not to include this task in our main analyses.

All subjects underwent a visual localizer comprising images of human faces, animal (faces with bodies), places, objects, logos, false fonts, English and Spanish words, numbers and Persian numbers. Each image was presented for 400 ms, with an inter-stimulus time interval of 500 ms. During the stimuli presentation, the patient was asked to pay attention to the center of the screen where a dot that would randomly change color (from red to blue) was displayed. The patient was asked to respond using a keyboard (“press 1”) when the dot changed color.

This session was preprocessed similarly to the main task presented in the manuscript and univariate testing was performed to identify face-selective and human face-selective sites (permutation testing, FDR corrected). For face-selective sites, the human face and animal categories were pooled together, while all other categories formed the non-face category. The time window considered for this analysis was amended to account for shorter presentation duration to [100 400]ms. The results of this analysis are presented in Supplementary Table 6 and Supplementary Discussion 6.

### Temporal distribution of face information

Building on previously published methods^71^, we implemented a technique to estimate the onset of the task-induced trial-by-trial HFB response on each site. Importantly, this analysis was performed on the non-smoothed data to eliminate any confound associated with temporal smoothing. For each trial of one category, we normalize the signal with respect to peak amplitude and implement a sliding window with 30 ms bins with 28 ms overlap. We then estimate the signal average and standard deviation in a baseline time window of [−200 0]ms before onset (averaged across trials) and identify 25 consecutive bins, in which the average HFB power exceeds the baseline average plus one standard deviation. This criterion allows us to identify the task-induced signal as opposed to more transient pathological activity or artifactual spiking. The earliest time point of the first bin in this sequence is marked as the signal onset for a specific trial. In the case that 25 consecutive bins surpassing the baseline threshold are not found, we exclude that trial from further analysis. In the present work, we calculate the median over trial-by-trial ROL estimates in order to assign singular ROL values for specific sites. Sites for which a ROL value could not be obtained in 50% of the trials or more were discarded from the analysis.

We investigated whether the response onset to human faces is related to the site’s anatomical position or selectivity. To this end, we computed the ROL of the HFB amplitude generated by human face stimuli on human face-selective sites. The obtained ROL values (*n* = 40) were then correlated (Spearman correlation) with the sites’ anatomical position (the “*y*”-coordinate in MNI space, estimating how posterior or anterior in the TC a site is). Similarly, the ROL values for human face-selective sites were correlated (Spearman correlation) with the site’s selectivity to human faces. The same analysis was performed for task-active sites (*n* = 94) for comparison. For illustration, we computed the average HFB traces on sites with *y* < −45, −45 < *y* < −35, and −35 < *y* for both face-selective and task-active sites. The boundaries chosen here are taken from a separate electrophysiological study focused on face-processing in the ventral TC^49^. Our ROL analyses are subject to imprecisions, due to temporal smoothing related to the time-frequency decomposition and to the averaging of the HFB signal in 30 ms bins. Hence our focus is on estimating relationships between ROL and anatomical positions. Absolute values of ROL should be considered with care as different techniques lead to different ROL values^72^. To this end, we estimated the same correlations but subtracting the ROL value from the most face-selective site within each subject. These results, plotted in Figs. 3b–d and 4b, c ensure that the reported correlations are not driven by specific subjects or systematic errors in timing estimation. Finally, we estimated potential group differences in ROL between face-selective and task-active sites using a Wilcoxon rank-sum test. To mitigate the effect of the posterior to anterior ROL gradient found on both groups of sites, we matched each face-selective site to its closest task-active site in terms of *y*-coordinate (considering a maximum distance of 2 mm) and performed a paired Wilcoxon signed-rank test. The same analysis was also performed within each subject.

### Effects of signal amplitude and slope on ROL

Our method performs response onset detection at the trial level, based on unsmoothed data. However, different parameters of the signal could affect the obtained ROL values, including noise, signal amplitude, and signal slope. In this section, we used semi-simulated data to investigate the effect of signal amplitude and slope on the obtained ROL values. The level of noise is the one that is naturally present in ECoG data.

The semi-simulated data used in this work have been designed for other work^73^ and are described in detail below. The data and code for generating the simulation are available open-source (https://github.com/JessicaSchrouff/Simulated_ECoG).

The original data was recorded from subject S1, during a 5-min wakefulness rest period, with eyes closed. Sites assessed as “pathological” by medical doctors were discarded from further analysis. A fake experimental design was simulated: two conditions, “A” and “B”, presented at random every 1.9 s. The stimuli are further assumed to last for 1 s. This yielded 146 “stimuli”, 73 for each category.

Signal preprocessing was performed with specific ECoG routines (github/LBCN/Preprocessing_Pipeline) using Matlab (www.mathworks.com) and SPM12 (www.fil.ion.ucl.ac.uk/spm). First, the data were converted to SPM format and downsampled to 1 kHz. The continuous signal was filtered for line noise and harmonics (stop-band: 57–63 Hz, 117–123 Hz, and 177–183 Hz) and an automatic quality assessment identified “noisy” or “spiky” sites based on their variance and number of “jumps” (i.e., signal derivative > 100 µV), leaving 38 “good” sites. The data was re-referenced to the average of all good channels before being epoched in the [−400, 1400]ms window around “onset” and baseline corrected using the [−400, 0]ms window. Epochs displaying flat segments of >4 ms or “jumps” >100 µV were discarded from further analysis. The signal was then decomposed using a five-wavelets decomposition in the 70–170 Hz frequency band (step: 10 Hz, avoiding 120 Hz) to estimate HFB power. The time-frequency signal was *z*-scored based on the pooled baselines of all events in the [−300, 0]ms window before onset to avoid edge effects and smoothed in the [−200, 1200]ms window after onset by a 50 ms Gaussian window. Epochs displaying *z*-scores >8 were discarded, leaving 60 trials for condition “A” and 56 for “B”. This preprocessing procedure is very similar to the one used in the main part of the work, including bad channel rejection.

We then simulated signals according to the “fake” experimental design. All modifications of data structure were performed on the preprocessed data to avoid an effect of the preprocessing on the obtained results. To simulate neural signal, a ramp window was added to all epochs of condition “A” starting 0 ms after “onset” with a slope of 3 until 500 ms, on all “good” sites. The amplitude of the signal in condition “A”was varied by modifying the SNR between trials “A” and “B”. Hence, varying signal amplitude is strongly correlated with varying the selectivity of sites to condition “A”. The amplitude of the signal in the ramp window was computed based on a desired SNR on each site:

1 $$X_{{\mathrm{A}},{\mathrm{eff}}} = X_{\mathrm{A}} + {\mathrm{SNR}}_{{\mathrm{in}}} \times {\mathrm{std}}\left( {\bar X_{\mathrm{B}}} \right)$$

where *X*_A,eff_ represents the amplitude of the effective simulated signal for condition “A” trials, *X*_A_, the amplitude of the signal for trials “A”, SNR_in_, a fixed number representing the desired SNR, and $\bar X_{\mathrm{B}}$, the average trace of “B” trials. SNR_in_ was varied from 2 to 10 by steps of 0.5. In our real dataset, the distribution of estimated SNR varies from −2 to 17 on human face-selective sites, with only three sites with SNR > 10.

To estimate the effect of signal slope on the ROL results, we performed the same simulation but normalizing the amplitude of the signal in each trial before ROL detection (L_inf_ -norm, i.e., dividing by the maximum amplitude). This simulated dataset hence varies the slope of the signal, but not the amplitude (set to maximum of 1).

For each SNR level, ROL detection is performed at the trial level, for the “A” trials on each “good” site. All parameters are identical to the technique reported above. Trials with <50% of detected onsets were excluded from the results. This analysis was performed on both the un-normalized (i.e., varying amplitude at fixed slope) and the normalized (i.e., varying slope at fixed amplitude) simulated data. The results are presented in Supplementary Discussion 8 and Supplementary Fig. 4.

### Causal importance of TC sites for face perception

A set of electrical brain stimulations (EBS) was performed on seven of the eight subjects in addition to the experimental task (but not concurrently). The sites of stimulations were chosen based on a priori knowledge about their HFB responses to face and non-face stimuli, as assessed by the univariate analysis of a localizer task. During the procedure, face-selective and task-active sites were stimulated with electrical charge while the subjects were instructed to look at various real-world face stimuli (persons at the bedside). Across the seven subjects, the instructions given included (A) looking at the face, (B) looking at the lips, (C) looking at the nose, (D) looking at self in the mirror, (E) looking at a cartoon face on a sheet of paper, or (F) close eyes and imagine a face. Please note that these clinical procedures diverge from stimulus-locked electrical perturbation experiments. Between 3 to 8 mA (depending on the excitability of the stimulated site) were delivered at a duration ranging from 1 to 3 s at 50 Hz frequency and 200 μs pulse width, of a square wave electrical waveform in unipolar (subject S8) or bipolar montage (S8 and other subjects). In unipolar montage, a TC site and a remote cortical reference site were stimulated whereas in the bipolar montage, a pair of adjacent electrodes were stimulated. Sham stimulations were also administered at 0 mA. Continuous EEG monitoring showed no after discharges or epileptic activity during the sessions. Verbal reports were collected following each stimulation. We recognized an electrode as a catalyst in face perception change if (A) the stimulation of this electrode yielded a face-specific change; (B) the stimulation of this electrode paired with any other electrode still yielded a change; and (C) the result of the stimulation and its resulting face-perception change was replicable across multiple stimulation trials. In addition, in subject S8, the estimated cortical area affected by the stimulation on electrode 3 was calculated using the relationship between the estimated charge per trial and the cortical area affected^54^. The charge deposited per trial (μC) was calculated as a product of the pulse width (ms), current (mA), frequency (Hz), and duration (s) of stimulation for each trial; then, we estimated the cortical area (mm^2^) affected by the stimulation as a function of the charge deposited per trial (μC) according to the methods described in a previous publication^54^. Furthermore, using a one-tailed *z-*score test, we tested whether the proportion of sites that induced distortions in face perception where significantly different in the posterior versus mid fusiform gyrus across all subjects.

### Statistical testing

Throughout this work, statistical testing was performed using non-parametric permutations. When suited, the tests were paired (e.g., when comparing two conditions in terms of ROL value on the same set of sites, or when comparing the HFB response of a stimulus category to its baseline). A minimum of 1000 permutations was performed, to ensure a good estimation of the null distribution. FDR or Bonferroni correction was applied when multiple comparisons tested for the same effect (e.g., testing for human face selectivity on each site). Significance was determined at *p* < 0.05, after correction if applicable. Population statistics for the decoding models I–IV was performed using Wilcoxon signed-rank tests^69^.

### Reporting summary

Further information on research design is available in the Nature Research Reporting Summary linked to this article.

## Supplementary information

Supplementary Information

Description of Additional Supplementary Files

SUPPLEMENTARY MOVIE 1

Reporting Summary

## Data Availability

Due to the presence of patient identifying information in the data, we cannot release the presented recordings.

## Code availability

All analyses were performed in Matlab (www.mathworks.com). Preprocessing and univariate analyses were performed based on SPM (http://www.fil.ion.ucl.ac.uk/spm/) and in-house routines available at https://github.com/LBCN-Stanford/Preprocessing_pipeline. ROL in-house codes are available on Github at https://github.com/LBCN-Stanford/. Multivariate analyses were performed using a development version of PRoNTo^30,68^. This code will be released as PRoNTo v3 and be available at http://www.mlnl.cs.ucl.ac.uk/pronto/. The code to build semi-simulated data is available at https://github.com/JessicaSchrouff/Simulated_ECoG, along with the rest data from subject S1 used to generate the noise structure.
