# Supplementary material for: Fast temporal dynamics and causal relevance of face processing in the human temporal cortex
Source: Nat Commun. 2020 Jan 31;11:656. doi: 10.1038/s41467-020-14432-8 (PMC6994602; doi:10.1038/s41467-020-14432-8)
Supplement: Supplementary file 1 — Supplementary Information [file 41467_2020_14432_MOESM1_ESM.docx]

**Fast temporal dynamics and causal relevance of face processing in the human temporal cortex**

Schrouff et al.

**SUPPLEMENTARY INFORMATION**

**Supplementary figures:**

**
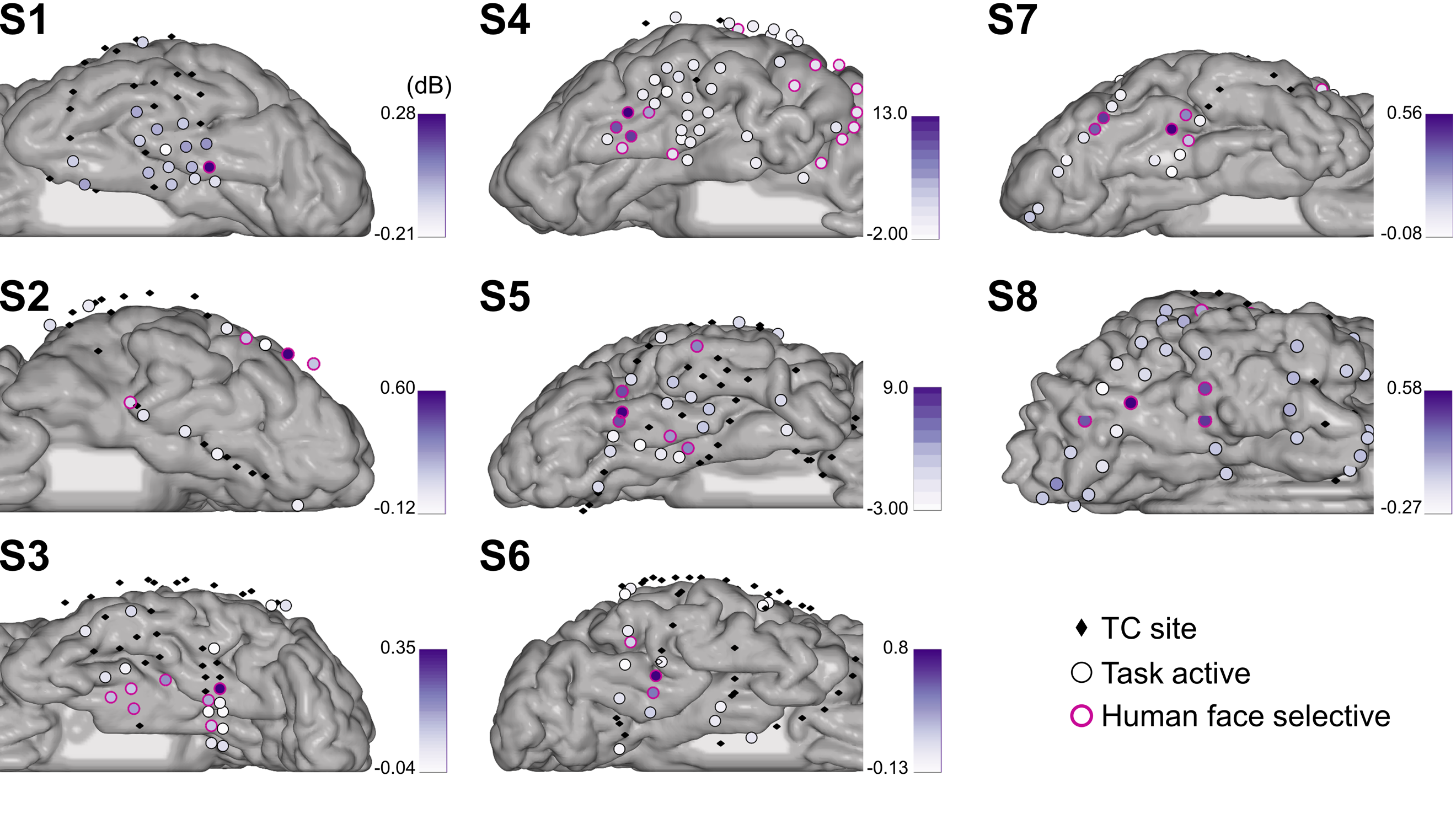
**

**Supplementary Figure 1: Human face selective sites, within subjects.** For each subject, the sites are represented on the individual cortex map. Sites represented by black diamonds were not assessed as task active. The first three subjects have left hemisphere implantation, while subjects S4 to S8 have right implantation. Sites displayed by circles were assessed as task active. Among those, sites highlighted by a pink rim were further assessed as human face selective. The human face selectivity of each site (in dB) is displayed via a color-coded fill of the task active sites.

**
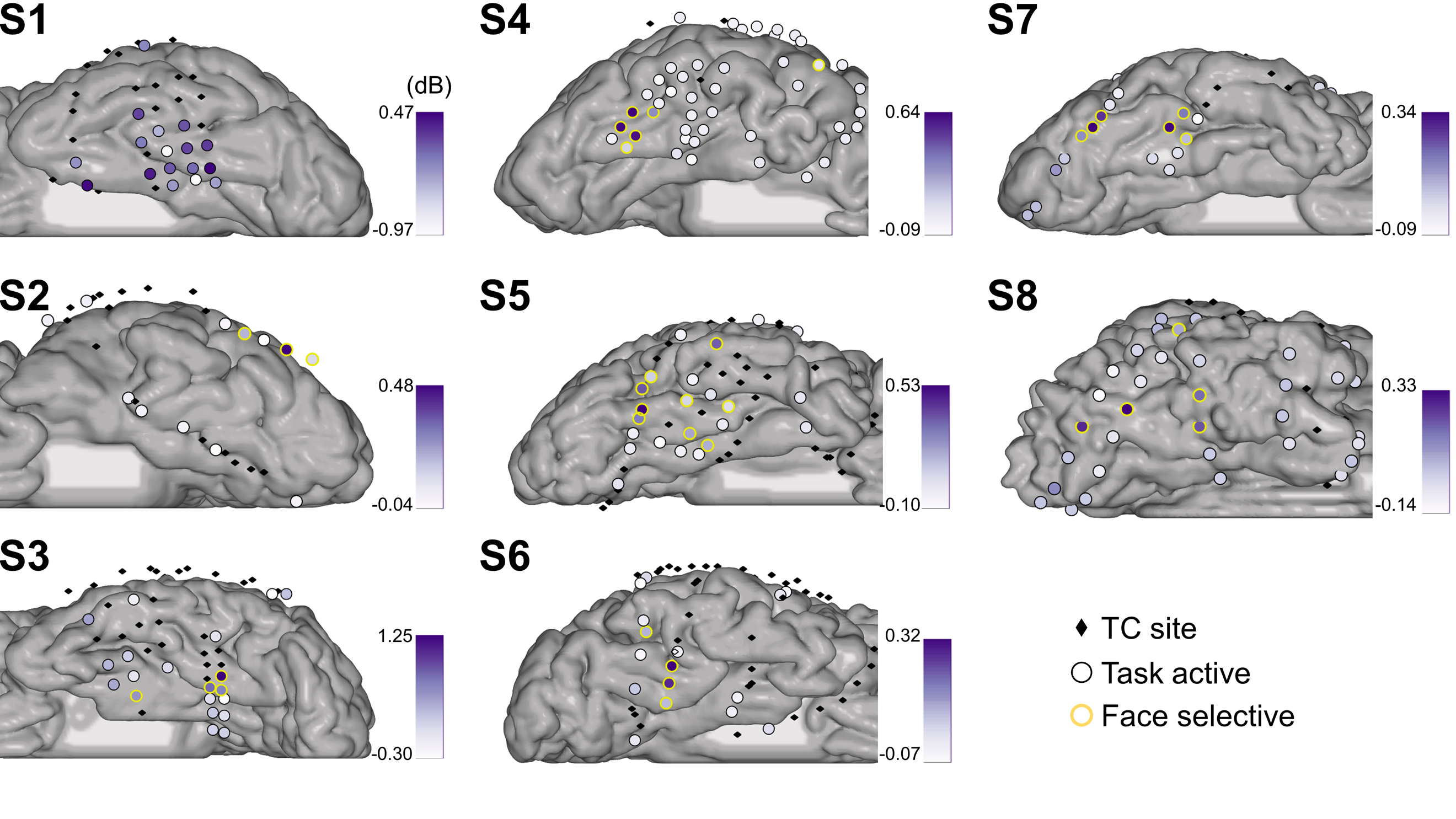
** **Supplementary Figure 2: Face selective sites, within subjects.** For each subject, the sites are represented on the individual cortex map. Sites represented by black diamonds were not assessed as task active. The first three subjects have left hemisphere implantation, while subjects S4 to S8 have right implantation. Sites displayed by circles were assessed as task active. Among those, sites highlighted by a yellow rim were further assessed as face selective (four face subcategories pooled). The face selectivity of each site (in dB) is displayed via a color-coded fill of the task active sites.


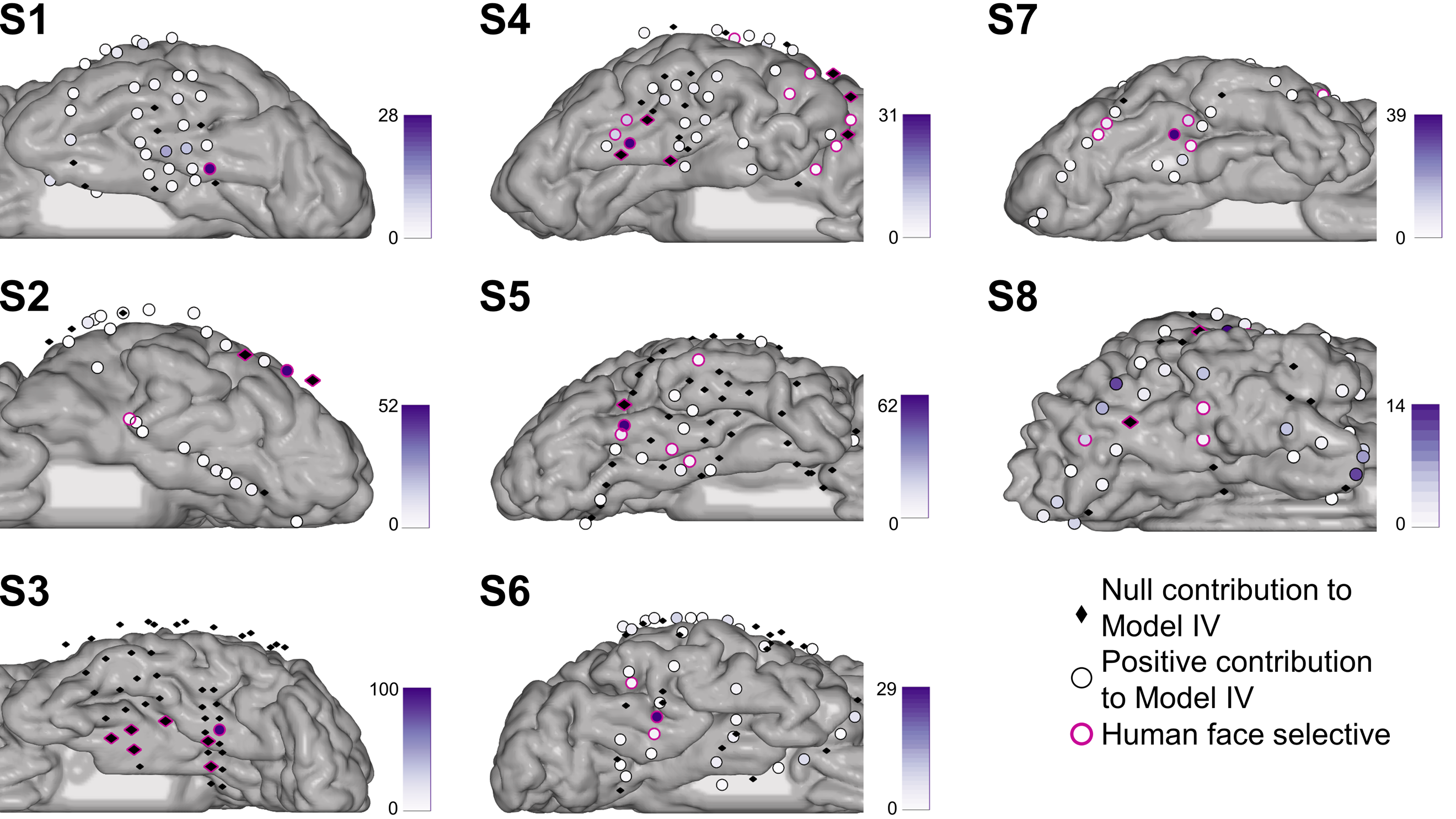
 **Supplementary Figure 3: Individual plots of site contribution to Model IV.** For each subject, the sites are displayed on the individual cortex mesh. Sites represented by black diamonds have a null contribution to Model IV. Sites represented by a circle have a positive contribution to Model IV, the amplitude of their contribution being color-coded using a purple fill. Sites assessed as human face selective by our univariate analysis are highlighted with a pink rim.


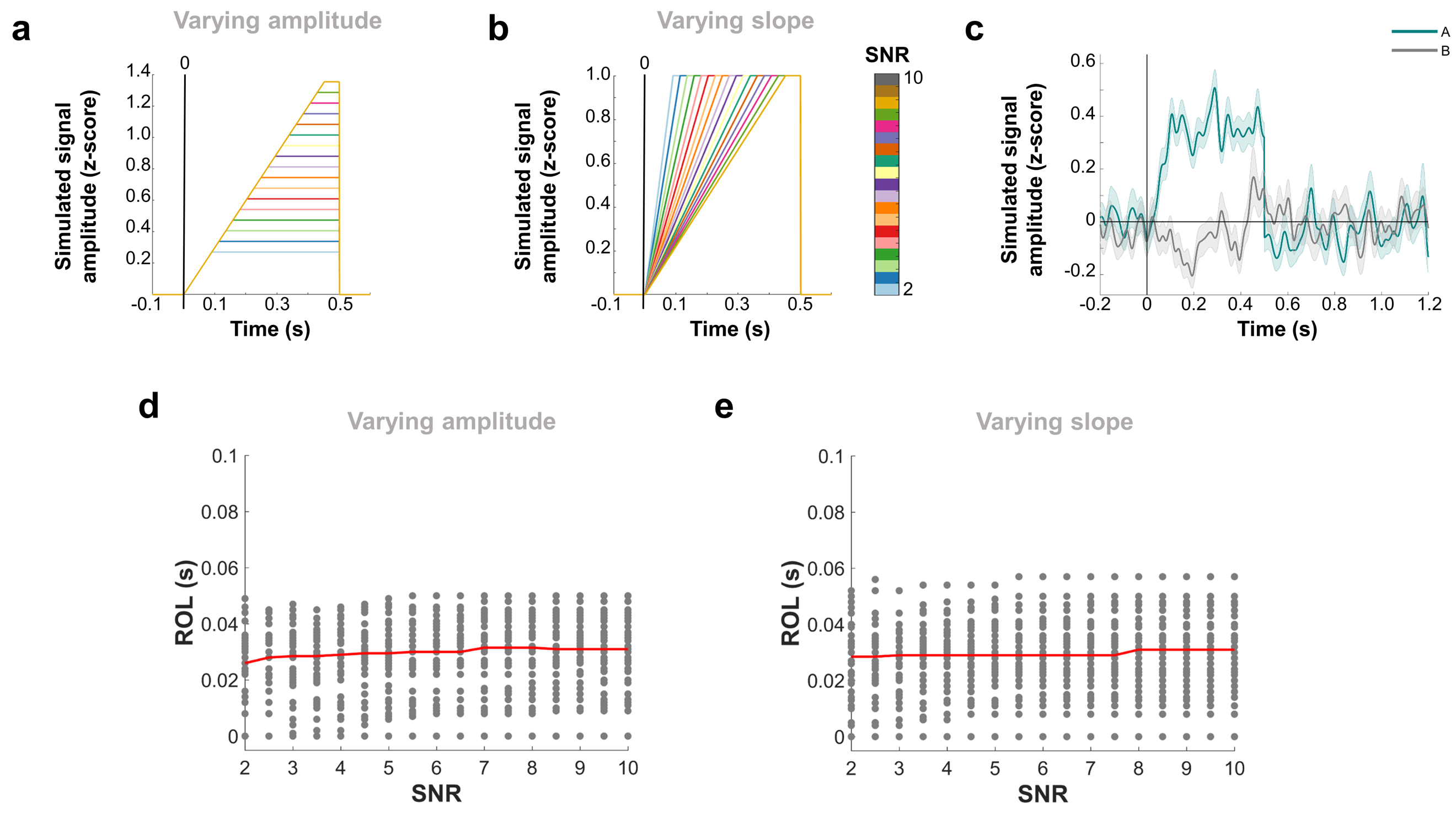


**Supplementary Figure 4: Effect of signal amplitude and of slope on ROL. (a)** Simulated ramp signals, for varying imposed SNR, for an example site. The onset time is 0ms after ‘onset’. In the present case, the slope is fixed and the amplitude varies with imposed SNR. **(b)** Simulated ramp signals after normalizing by the maximum amplitude (i.e. L_inf_-norm), for the same example site. In this case, the slope varies but the maximum amplitude is fixed at 1. **(c)** Example obtained semi-simulated signals for one site, at imposed SNR = 3. The average trace for condition ‘A’ is displayed in green (with shaded standard error), and condition ‘B’ (unused in this work), in grey. The amount of noise represented is the on-going resting activity from the recorded data. **(d)** When varying amplitude at fixed slope (i.e. un-normalized signals), SNR does not affect significantly the detected ROL across sites (ρ = 0.0613, p = 0.1198). **(e)** Similar results are obtained when varying slope at fixed amplitude, i.e. normalizing the signals (ρ = 0.0245, p = 0.5335). Please note that the effect of noise is displayed by the variability in ROL across sites for each SNR.

**Supplemental Information:**

**Supplementary Table 1: Demographics.**

| Subject # | Age | Gender | Native Language | Language Lateralization | Handedness | Type of epilepsy | Side of implantation (Number of electrodes) | IQ | Duration of epilepsy |
| --- | --- | --- | --- | --- | --- | --- | --- | --- | --- |
| 1 | 47 | male | English | Bilateral | left | Right temporal lobe epilepsy | left (64) | 74 | 13 years |
| 2 | 44 | male | Spanish | N/A | right | Left Temporal lobe epilepsy | left (106) | N/A | 41 years |
| 3 | 23 | male | English | Left | right | Left temporal oligodendroglioma | left (125) | 100 | 6 years |
| 4 | 68 | male | Korean | N/A | right | Right temporal lobe | right (96) | N/A | 32 years |
| 5 | 65 | female | English | Right | right | Right temporal lobe epilepsy | right (110) | 113 | 4 years |
| 6 | 35 | male | German; fluent in English | Left | right | Right fronto-temporal lobe | right (126) | 129 | 18 years |
| 7 | 36 | female | English | N/A | right | Right temporal lobe epilepsy | right (128) | 102 | 6 years |
| 8 | *** | male | English | *** | | | | | |

*** Because we are enclosing a video of this subject, we are keeping his information confidential.

**Supplementary Discussion 2: Frequency information for faces**

Supplementary Table 2 suggests that other frequency bands carry (human) face information. However, the MKL model strongly prefers the HFB to discriminate human faces from non-faces, in all subjects (Figure 2b). This result suggests that the human face information carried by other bands is either weaker or highly correlated with the HFB human face information (as the MKL model only selects non-correlated information). Similarly, the MKL model combining ERPs with HFB assigned a large contribution to the HFB signals (average across subjects: 85.15%). Therefore, using the amplitude of the HFB power as an index of neural population activity for investigating (human) face distribution over the TC seems reasonable.”

**Supplementary Table 2: Task active and (human) face selective sites in each frequency band.** The number of identified task active, face selective and human face selective sites is displayed, summed over all subjects.

| Band | Task active | Face selective | Human Face selective |
| --- | --- | --- | --- |
| θ | 122 | 2 | 2 |
| α | 104 | 5 | 7 |
| β_1_ | 98 | 17 | 25 |
| β_2_ | 80 | 9 | 23 |
| γ | 114 | 21 | 30 |
| HFB | 193 | 37 | 48 |

**Supplementary Table 3: TC sites and category-specific responses.** For each participant, the number of sites located in the TC is displayed. Sites were excluded from further analysis if they exhibited epileptic activity or excessive noise artifacts. The number of task active (3^rd^ column), face selective (4^th^ column) and human face selective (last column) sites are also displayed. The total for each column is displayed at the bottom of the table.

| Patient | TC sites | Task active | Face selective | Human face selective |
| --- | --- | --- | --- | --- |
| S1 | 39 | 17 | 0 | 1 |
| S2 | 29 | 12 | 3 | 4 |
| S3 | 50 | 20 | 4 | 7 |
| S4 | 49 | 45 | 6 | 15 |
| S5 | 59 | 23 | 9 | 6 |
| S6 | 55 | 16 | 4 | 3 |
| S7 | 30 | 21 | 6 | 6 |
| S8 | 46 | 36 | 5 | 6 |
| **Total** | **357** | **190** | **37** | **48** |

**Supplementary Discussion 4: Low-level image features**

*Stimulus features*

There were no significant differences in spatial information when comparing the 11 categories, pair-wise (one-way ANOVA, p-values = 0.9999). Mean luminance was significantly different between human faces and non-faces (p=9.93e-09, n= 200), mammal faces and non-faces (p=6.3e-08, n=200), bird faces and non-faces (p=4.5e-04, n=200) and marine faces and non-faces (p=6.2e-07, n=200). No significant differences were found between human and mammal faces (p=0.88, n= 25), human and bird faces (p=0.15, n=25), human and marine faces (p=0.71, n=25), mammal and bird faces (p=0.67, n=25), mammal and marine faces (p=0.99, n=25) and bird and marine faces (p=0.84, n=25).

*Univariate analysis*

When comparing low and high luminance faces, no site displayed a significant difference between the two categories (5 before FDR correction, permutation test, Supplementary Table 4). These results need to be considered with care, as these negative results cannot completely reject the null hypothesis (they might be under-powered). However, the obtained results suggest that there is no effect of luminance on our main findings.

**Supplementary Table 4: Effect of luminance on face sites.** P-values (permutation test) of univariate analyses comparing low luminance face stimuli with high luminance face stimuli.

| \| Number \| p-value(High vs. Low luminance face) \| \| --- \| --- \| \| 01. \| 0.235 \| \| 02. \| 0.217 \| \| 03. \| 0.030 \| \| 04. \| 0.218 \| \| 05. \| 0.046 \| \| 06. \| 0.315 \| \| 07. \| 0.354 \| \| 08. \| 0.302 \| \| 09. \| 0.112 \| \| 10. \| 0.371 \| \| 11. \| 0.227 \| \| 12. \| 0.029 \| \| 13. \| 0.367 \| \| 14. \| 0.458 \| \| 15. \| 0.088 \| \| 16. \| 0.160 \| \| 17. \| 0.272 \| \| 18. \| 0.422 \| \| 19. \| 0.347 \| \| 20. \| 0.306 \| \| 21. \| 0.352 \| \| 22. \| 0.064 \| \| 23. \| 0.399 \| \| 24. \| 0.109 \| \| 25. \| 0.199 \| \| 26. \| 0.332 \| \| 27. \| 0.467 \| | \| Number \| p-value(High vs. Low luminance face) \| \| --- \| --- \| \| 28. \| 0.366 \| \| 29. \| 0.371 \| \| 30. \| 0.307 \| \| 31. \| 0.060 \| \| 32. \| 0.408 \| \| 33. \| 0.134 \| \| 34. \| 0.399 \| \| 35. \| 0.416 \| \| 36. \| 0.297 \| \| 37. \| 0.220 \| \| 38. \| 0.393 \| \| 39. \| 0.346 \| \| 40. \| 0.271 \| \| 41. \| 0.337 \| \| 42. \| 0.352 \| \| 43. \| 0.228 \| \| 44. \| 0.342 \| \| 45. \| 0.341 \| \| 46. \| 0.158 \| \| 47. \| 0.066 \| \| 48. \| 0.306 \| \| 49. \| 0.001 \| \| 50. \| 0.046 \| \| 51. \| 0.184 \| \| 52. \| 0.352 \| \| 53. \| 0.444 \| \| 54. \| 0.221 \| |
| --- | --- | --- | --- | --- | --- | --- | --- | --- | --- | --- | --- | --- | --- | --- | --- | --- | --- | --- | --- | --- | --- | --- | --- | --- | --- | --- | --- | --- | --- | --- | --- | --- | --- | --- | --- | --- | --- | --- | --- | --- | --- | --- | --- | --- | --- | --- | --- | --- | --- | --- | --- | --- | --- | --- | --- | --- | --- | --- | --- | --- | --- | --- | --- | --- | --- | --- | --- | --- | --- | --- | --- | --- | --- | --- | --- | --- | --- | --- | --- | --- | --- | --- | --- | --- | --- | --- | --- | --- | --- | --- | --- | --- | --- | --- | --- | --- | --- | --- | --- | --- | --- | --- | --- | --- | --- | --- | --- | --- | --- | --- | --- | --- | --- |

**Supplementary Table 5: Relevance of face and non-face sites in decoding**. The first column displays the balanced accuracy for Model I, with the p-value that it differs from the distribution defined by the ‘random set’ models. The second column displays the performance of the same model when discarding human face selective sites (Model II), with the p-value that it differs from the ‘random set’ models. Column 3 displays the balanced accuracy of the same model when removing all task active sites (‘TC-task’, Model IIIa). Column 4 displays the results when random subsets of sites are discarded (‘random set’, median across 499 models). Column 5 displays the correlation between the accuracy of ‘random set’ models and the proportion of human face sites included in the model. Significant results are displayed in bold (permutation test, p<0.05, corrected). Results displayed in italic show a trend (permutation test, p<0.05 uncorrected).

| Patient | ‘TC’ (I, in %) | ‘TC-face’ (II, in %) | ‘TC-task’ (IIIa, in %) | ‘random set’ (III, in %) | Corr(F,acc) |
| --- | --- | --- | --- | --- | --- |
| S1 | **68.00** (p=0.4980) | 61.71 (**p=0.0020**) | **68.86** | 68.00 | N.a. |
| S2 | **85.54** (p=0.3000) | 62.82 (p=0.0680) | **80.96** | 84.64 | **0.4697** |
| S3 | **75.22** (p=0.2340) | 58.27 (**p=0.0060**) | **69.11** | 72.52 | **0.2441** |
| S4 | **94.98** (p=0.7620) | **69.48** (**p=0.0020**) | **94.12** | 95.58 | **0.2060** |
| S5 | **98.08** (p=0.1340) | 53.89 (**p=0.0020**) | **96.33** | 98.00 | **-0.2144** |
| S6 | **80.10** (p=0.6180) | **73.41**(**p=0.0020**) | **69.91** | 80.55 | *0.0971* |
| S7 | **85.86** (p=0.6080) | 64.16 (**p=0.0020**) | **83.56** | 86.82 | **0.4119** |
| S8 | **94.10** (p=0.0580) | 60.45 (**p=0.0020**) | **79.93** | 91.59 | **0.2611** |

**Supplementary Discussion 6: Univariate results on visual localizer**

Supplementary Table 6 displays the number of sites identified as face and human face selective on this task, as well as the number of sites commonly identified in both tasks. It reveals that 60.53% of sites identified on the main task were reported as face selective in both tasks, while 58.33% of sites were reported as human face selective in both tasks. Although the two tasks have fundamentally different contrasts (the visual localizer contains more images of words than of pictures, and the animal category contains both face and body), the overlap of selected face and human face sites is significant. It is therefore unlikely that our reported results are driven by task specific features.

**Supplementary Table 6**: **Consistence of face and human face selective sites identification across tasks.** Number of face and human face selective sites identified on a visual localizer task (‘Loc’), and identified on both our main task and the visual localizer (‘both’).

| Patient | Face selective (Loc) | Face selective (both) | Human face selective (Loc) | Human face selective (Both) |
| --- | --- | --- | --- | --- |
| S1 | 6 | 0 | 3 | 1 |
| S2 | 3 | 2 | 2 | 2 |
| S3 | 5 | 2 | 2 | 2 |
| S4 | 2 | 0 | 8 | 6 |
| S5 | 9 | 5 | 16 | 5 |
| S6 | 10 | 4 | 6 | 3 |
| S7 | 10 | 7 | 7 | 5 |
| S8 | 7 | 3 | 5 | 4 |
| **Total** | **52** | **23** | **49** | **28** |

**Supplementary Table 7: Distributed versus sparse anatomical prior.** The first column copies the accuracy from Model I (balanced accuracy, in %), for ease of comparison. The second column displays the balanced accuracy (in %) obtained from a sparse Multiple Kernel Learning model. The third column displays the sparsity of Model IV (averaged across folds), a measure close to 0 representing a low number of sites selected while a value close to 1 represents all sites being selected. Column four displays the correlation (with p-value) between site contributions to the model and the human face selectivity as identified by the univariate test ‘human faces versus pooled non-faces’.

| Patient | ‘TC’ (I, in %) | sMKL (IV, in %) | Sparsity | Corr(beta,uni) |
| --- | --- | --- | --- | --- |
| S1 | **68.00** | **76.95** | 0.33 | **0.4426** (p=0.0048) |
| S2 | **85.54** | **90.71** | 0.50 | **0.6714** (p<0.0001) |
| S3 | **75.22** | **80.07** | 0.44 | **0.6773** (p<0.0001) |
| S4 | **94.98** | **96.00** | 0.33 | **0.7635** (p<0.0001) |
| S5 | **98.08** | **96.15** | 0.02 | **0.8681** (p<0.0001) |
| S6 | **80.10** | **77.57** | 0.40 | **0.7272** (p<0.0001) |
| S7 | **85.86** | **90.86** | 0.37 | **0.6855** (p<0.0001) |
| S8 | **94.10** | **91.78** | 0.41 | -0.1405 (p=0.3517) |

**Supplementary Discussion 8: Effects of signal amplitude and slope on ROL**

Examples of simulated ramp signals and obtained semi-simulated signals are presented in Supplementary Figure 4 (a-c), on one example site. Supplementary Figure 4a displays the simulated signal obtained for different SNRs when varying amplitude while Supplementary Figure 4b displays the simulated signal obtained for different SNRs when varying slope (i.e. normalizing the signal).

The ROL-SNR relationships are displayed in Supplementary Figure 4 for the un-normalized (d) and normalized signals (e). When varying amplitude at fixed slope, correlation between detected onset and estimated SNR was close to 0 (ρ = 0.0613, p = 0.1198) across all 38 sites (displayed as grey dots on the scatter plot). When varying slope at fixed amplitude, similar results were found (ρ = 0.0245, p = 0.5335). In either case, the average ROL varies at most by 6 (un-normalized) or 12 (normalized) ms between SNR = 2 and SNR =10. This scale of variation is much smaller than the effect reported between posterior and anterior sites in the main text.

The proposed ROL detection method is independent of signal amplitude and slope. In addition, the size of a potential bias is limited and much inferior to the effect reported in the main text. In conclusion, it is unlikely that the reported effects result only from biases in our ROL method.

**Supplementary Table 9: Subject responses during electrical stimulation trials.** For each subject, the stimulations leading to changes in subjective face perception are displayed, with the MNI location of the stimulated sites, current (Curr) and duration (Dur) of the stimulation and the (truncated) verbal report of the subject. Human face sites are highlighted in light pink.

|  | Stimulation Pair | | | | | | Curr (mA) | Dur (s) | Patient reporting changes in face perception after stimulation |
| --- | --- | --- | --- | --- | --- | --- | --- | --- | --- |
|  | x | y | z | x | y | z |  |  |  |
| S1 | -28 | -49 | -23 | -24 | -49 | -19 | 6 | 1.5 | "[The face] changed a little...Just that left side of her face changed...It looks like my wife's cousin or something" |
|  | -28 | -49 | -23 | -28 | -55 | -22 | 6 | 2.5 | "[The face] looks like my neighbor's wife or something on one side." |
|  | -28 | -49 | -23 | -35 | -47 | -27 | 6 | 1.8 | "[The face] was like a face in a movie I've seen but I can't remember the movie, just the half part [changed]… it changed to a face" |
|  | -35 | -47 | -27 | -44 | -47 | -30 | 6 | 1.6 | "About the same thing. It looks like a person I've seen in a movie." |
|  | -35 | -47 | -27 | -37 | -54 | -25 | 6 | 1.7 | "Very little [changed], not too much...Not as much as the previous one...it was less." |
|  | -23 | -56 | -18 | -28 | -55 | -22 | 6 | 1.5 | "That side [the pt's right side] changed… It looked like that side [PT's right side] turned into somebody else." |
| S2 | No stimulation performed | | | | | | | | |
| S3 | Stimulation was performed, but no changes in face perception observed | | | | | | | | |
| S4 | 38 | -62 | -24 | 35 | -56 | -25 | 3 | 1 | "His right eye… looks like some stone." |
|  | 38 | -62 | -24 | 39 | -53 | -26 | 4 | 2 | "Her right eye looks like a dance motion." |
|  | 38 | -62 | -24 | 42 | -50 | -27 | 4 | 1 | "No change, but there was some twist [gesturing to bridge of nose]." |
|  | 42 | -58 | -25 | 39 | -53 | -26 | 4 | 1 | "[I] was looking at my eye and he said that initially it looked like a circle but then it turned into a rectangular shape." |
|  | 42 | -58 | -25 | 39 | -53 | -26 | 4 | 2 | "The right eye looked normal and then it twisted." |
|  | 42 | -58 | -25 | 39 | -53 | -26 | 3 | 1 | "His right eye… some square… a little square" Translator: "I think he's talking about a diamond shape." |
|  | 42 | -58 | -25 | 39 | -53 | -26 | 3 | 2 | "My right eye [in the mirror], a little more bigger. And left eye, a little smaller." |
|  | 45 | -56 | -26 | 42 | -50 | -27 | 3 | 1 | "The chin looks a little droopy." |
| S5 | Stimulation was performed, but no changes in face perception observed | | | | | | | | |
| S6 | Stimulation was performed, but no changes in face perception observed | | | | | | | | |
| S7 | Stimulation was performed, but no changes in face perception observed | | | | | | | | |

| S8 | 39 | -40 | -30 | 36 | -66 | -18 | 8 | 2 | "Your eye on your right side changed a little bit." |
| --- | --- | --- | --- | --- | --- | --- | --- | --- | --- |
|  | 39 | -40 | -30 | 36 | -66 | -18 | 8 | 2 | "Your eye did. " |
|  | 39 | -40 | -30 | 36 | -66 | -18 | 8 | 2 | "I don’t recognize it. It turned into a cartoon. Your right eye side. The right eye. It’s something I don’t recognize. " |
|  | 39 | -40 | -30 | 36 | -66 | -18 | 8 | 2 | "I see part of a cartoon. I don’t see it really good images in my mind, it’s just like a brain image but not a mind image. I saw this distorted face. The side… Part of the upper. " |
|  | 39 | -40 | -30 | 36 | -66 | -18 | 8 | 2 | "Right eye was starting to distort… The right eye is reminding me of Lassie back when I was a kid. It was a tv show and the dog Lassie. Just the right eye part. " |
|  | 36 | -66 | -18 | 37 | -1 | 59 | 8 | 2 | " [Doctor's right eye] becomes somebody’s else, I mean, somebody else’s...I recognized it...it looked familiar." |
|  | 36 | -66 | -18 | 37 | -1 | 59 | 8 | 2 | "[My left] eye changed [in the mirror]...The eyeball is somebody else’s...It was just the eye change, but I still recognized myself." |
|  | 36 | -66 | -18 | 42 | -65 | -20 | 8 | 1 | "[The doctor's lips] just kind of wiggled a little bit. It reminded me of somebody. Familiar in movies…Just the right hand side looked like somebody's. " |
|  | 36 | -66 | -18 | 42 | -65 | -20 | 8 | 1 | "Just for one eye [of the doctor changed] for about two seconds... I guess like the whole face." |
|  | 36 | -66 | -18 | 42 | -65 | -20 | 8 | 1 | "The right eye [of the cartoon the patient is seeing]. It like looked sideways." |
