# Supplementary material for: Fast temporal dynamics and causal relevance of face processing in the human temporal cortex
Source: Nat Commun. 2020 Jan 31;11:656. doi: 10.1038/s41467-020-14432-8 (PMC6994602; doi:10.1038/s41467-020-14432-8)
Supplement: Supplementary file 2 — Description of Additional Supplementary Files [file 41467_2020_14432_MOESM2_ESM.docx]

Description of Additional Supplementary Files

**Supplementary Movie 1:** Video of subject S8’s verbal responses during electrical stimulation procedure. The patient has consented to the use of his video in this publication.
