# Supplementary material for: Fast temporal dynamics and causal relevance of face processing in the human temporal cortex
Source: Nat Commun. 2020 Jan 31;11:656. doi: 10.1038/s41467-020-14432-8 (PMC6994602; doi:10.1038/s41467-020-14432-8)
Supplement: Supplementary file 4 — Reporting Summary [file 41467_2020_14432_MOESM4_ESM.pdf]

nature research

Corresponding author(s): Josef Parvizi

Last updated by author(s): 24/11/2019

Reporting Summary

Nature Research wishes to improve the reproducibility of the work that we publish. This form provides structure for consistency and transparency in reporting. For further information on Nature Research policies, see Authors & Referees and the Editorial Policy Checklist.

Statistics

For all statistical analyses, confirm that the following items are present in the figure legend, table legend, main text, or Methods section.

n/a	Confirmed	
☐	☒	The exact sample size (n) for each experimental group/condition, given as a discrete number and unit of measurement
☐	☒	A statement on whether measurements were taken from distinct samples or whether the same sample was measured repeatedly
☐	☒	The statistical test(s) used AND whether they are one- or two-sided<br>*Only common tests should be described solely by name; describe more complex techniques in the Methods section.*
☐	☒	A description of all covariates tested
☐	☒	A description of any assumptions or corrections, such as tests of normality and adjustment for multiple comparisons
☐	☒	A full description of the statistical parameters including central tendency (e.g. means) or other basic estimates (e.g. regression coefficient) AND variation (e.g. standard deviation) or associated estimates of uncertainty (e.g. confidence intervals)
☐	☒	For null hypothesis testing, the test statistic (e.g. F, t, r) with confidence intervals, effect sizes, degrees of freedom and P value noted<br>*Give P values as exact values whenever suitable.*
☒	☐	For Bayesian analysis, information on the choice of priors and Markov chain Monte Carlo settings
☒	☐	For hierarchical and complex designs, identification of the appropriate level for tests and full reporting of outcomes
☐	☒	Estimates of effect sizes (e.g. Cohen's d, Pearson's r), indicating how they were calculated

Our web collection on statistics for biologists contains articles on many of the points above.

Software and code

Policy information about availability of computer code

Data collection	Data were obtained at 1525.88 Hz through a 128-channel recording system (Tucker Davis Technologies, http://www.tdt.com) for the first seven subjects while a Nihon Kohden Technology system with simultaneous video monitoring was used to perform 1 kHz recordings in subject S8. The recordings were exported as text files (or EDF for S8) using their proprietary software for further import into Matlab and preprocessing pipeline.
Data analysis	All analyses were performed in Matlab (www.mathworks.com). Pre-processing and univariate analyses were performed based on SPM (http://www.fil.ion.ucl.ac.uk/spm/) and in-house routines available at https://github.com/LBCN-Stanford/Preprocessing_pipeline. ROL in-house codes are available on Github at https://github.com/LBCN-Stanford/. Multivariate analyses were performed using a development version of PRoNTo. This code will be released as PRoNTo v3 and be available at http://www.mlnl.cs.ucl.ac.uk/pronto/ (currently in beta testing phase). The code to build semi-simulated data is available at https://github.com/JessicaSchrouff/Simulated_ECoG, along with the rest data from subject S1 used to generate the noise structure.

For manuscripts utilizing custom algorithms or software that are central to the research but not yet described in published literature, software must be made available to editors/reviewers. We strongly encourage code deposition in a community repository (e.g. GitHub). See the Nature Research guidelines for submitting code & software for further information.

Data

Policy information about availability of data

All manuscripts must include a data availability statement. This statement should provide the following information, where applicable:

- Accession codes, unique identifiers, or web links for publicly available datasets
- A list of figures that have associated raw data
- A description of any restrictions on data availability

Due to the presence of patient identifying information in the data, we cannot release the presented recordings. This is stated in the main text.

Field-specific reporting

Please select the one below that is the best fit for your research. If you are not sure, read the appropriate sections before making your selection.

☒ Life sciences ☐ Behavioural & social sciences ☐ Ecological, evolutionary & environmental sciences

For a reference copy of the document with all sections, see nature.com/documents/nr-reporting-summary-flat.pdf

Life sciences study design

All studies must disclose on these points even when the disclosure is negative.

Sample size	8 subjects were included in this study, including 357 recording sites. Analyses were performed within or across subjects, as appropriate.
Data exclusions	Sites displaying excessive amounts of noise (whether pathological or not) were discarded from the analysis. This process is fully described in the methods.
Replication	The analyses were performed using univariate and multivariate techniques, including a framework as defined in Weichwald et al., 2015 as well as sparse methods. Our EBS procedure replicates our and other previous work on fusiform area stimulation.
Randomization	N.a.
Blinding	N.a.

Reporting for specific materials, systems and methods

We require information from authors about some types of materials, experimental systems and methods used in many studies. Here, indicate whether each material, system or method listed is relevant to your study. If you are not sure if a list item applies to your research, read the appropriate section before selecting a response.

Materials & experimental systems

n/a	Involved in the study	
☒	☐	Antibodies
☒	☐	Eukaryotic cell lines
☒	☐	Palaeontology
☒	☐	Animals and other organisms
☐	☒	Human research participants
☒	☐	Clinical data

Methods

n/a	Involved in the study	
☒	☐	ChIP-seq
☒	☐	Flow cytometry
☒	☐	MRI-based neuroimaging

Human research participants

Policy information about studies involving human research participants

Population characteristics	Subjects undergoing intracranial electroencephaloengraphy for the purpose of tracking drug-resistant epilepsy were included in this study on a volunteer basis.
Recruitment	We recruited subjects during their hospital stay for continuous monitoring of seizures. The study or the involvement of the subjects did not affect their clinical care and informed consent was obtained. While we excluded pathological and noisy recording sites, we are aware that this population of subjects is highly heterogeneous and does not represent the general population.
Ethics oversight	The study was approved by the Stanford IRB.

Note that full information on the approval of the study protocol must also be provided in the manuscript.
